# Anomalies in dye-terminator DNA sequencing caused by a natural G-quadruplex

**DOI:** 10.1371/journal.pone.0279423

**Published:** 2022-12-27

**Authors:** George S. Brush

**Affiliations:** Department of Oncology, Molecular Therapeutics Program, Barbara Ann Karmanos Cancer Institute, Wayne State University School of Medicine,Detroit, MI, United States of America; CNR, ITALY

## Abstract

A G-rich DNA sequence from yeast that can form a non-canonical G-quadruplex structure was cloned into a plasmid vector and subjected to Sanger sequencing using dye-labeled dideoxynucleotides. Two different effects were observed. In one, presence of the G4 sequence on the template strand led to incorrect incorporation of an A residue at an internal position in the G4 sequence. In the other, the nascent strand caused attenuation of the readout coincident with synthesis of the G-rich DNA. The two effects are novel examples of disruption in DNA synthesis caused by a G4 sequence. These results provide a new example of a DNA structure that could influence genomic stability in human cells.

## Introduction

G-quadruplex (G4) DNA is formed through Hoogsteen base pairing of guanines in a stacked planar arrangement with four DNA strands [1, 2]. Different configurations exist, in each case stabilized by biologically relevant monovalent cations [3]. While early studies mainly concerned single-stranded (ss) DNA, both inter- and intramolecular, there is evidence that intramolecular G4 structures form in naturally derived duplex DNA as well, at physiologic pH and in equilibrium with the normal DNA double strand helix [4]. Various studies have revealed that G4 elements are spread throughout the entire human genome, with enrichment in gene promoter regions [5–8]. Of considerable interest is the role that G4 DNA has in regulating transcription of particular genes, such as the *MYC* oncogene [9]. These findings have led to an interest in targeting certain G4 sequences for cancer therapeutic purposes [10], as have the earlier studies that revealed G4 sequences are found in telomeres [11, 12]. It has been suggested that G4 sequences are also found near origins of DNA replication in metazoans and could regulate their function [13–15]. However, the degree of association varies with the methodology employed to define human origins, as one recent study suggested little if any enrichment of G4 DNA at human origins [16] while another confirmed the correlation [17]. A comprehensive study using different DNA replication systems has further indicated that an Origin G-Rich Repeated Element capable of forming a G4 structure contributes to initiation of DNA replication and is found in most active origins near the initiation site [18].

Experiments with purified enzymes and templates have revealed that intramolecular G4 structures can inhibit DNA synthesis catalyzed by various DNA polymerases, including Klenow fragment, Sequenase (T7), T4, Taq, and a variety of replication, repair, and translesion polymerases from yeast and human cells [19–24]. As might be expected, in these events the G4 acts as a “roadblock” wherein DNA synthesis is arrested when the DNA polymerase encounters the G4 structure on the template strand. In the case of *S*. *cerevisiae* polδ, addition of the human WRN helicase allows replication through and past the G4 [22]. Additional studies have shown that other RecQ-type helicases can unwind G4 DNA, as can a number of other helicases including human FANCJ and *S*. *cerevisiae* Pif1 helicase [25–30]. Cells defective for individual helicases often show genetic instability; interestingly, the FANCJ DNA helicase is important for suppressing DNA deletions in regions containing canonical G4 DNA sequences [31]. This correlation supports the notion that G4 structures can lead to genomic instability. Further evidence from avian cells has indicated that polymerases REV1 and PrimPol are required for navigating G4 structures during DNA replication, thereby preventing epigenetic instability [32, 33]. It has also been shown that various translesion DNA polymerases can bypass a G4 template structure, although this event would lead to a deletion in the nascent strand [30].

As a first step in developing a screen to investigate G4 DNA management, a plasmid was generated containing a verified G4 DNA sequence identified in *S*. *cerevisiae* [34]. This sequence interfered with DNA synthesis as measured by Sanger sequencing [35] with dye-labeled dideoxynucleotides. In one case, the G4 structure affected DNA synthesis when it was located on the template strand; in the other, the effect was associated with the nascent strand. These observations indicate new mechanisms by which a G4 DNA structure can negatively influence DNA synthesis.

## Materials and methods

### Molecular cloning

ODNs (IDT) were designed to include G4 wt or variant sequences with endogenous flanking sequences (from yeast). Ends were designed to form overhangs when complementary oligomers were annealed so that the duplex could be inserted between BamHI and EcoRI in plasmid pRS412 [36] (see Table 1 for G4-1 and variants). The pG4-2 and pG4-2-v plasmids were constructed with the following ODNs:

G4-2-a, 5’-GATCCTGATTTGGA**GGG**TACGGT**GGG**TAATAA**GGG**AAGGTATC**GGG**ATT**GGGG**TAG-3’
G4-2-b, 5’-AATTCTACCCCAATCCCGATACCTTCCCTTATTACCCACCGTACCCTCCAAATCAG-3’
G4-2-v-a, 5’-GATCCTGATTTGGA**GCG**TACGGT**GCG**TAATAA**GCG**AAGGTATC**GCG**ATT**GGGG**TAG-3’
G4-2-v-b, 5’-AATTCTACCCCAATCGCGATACCTTCGCTTATTACGCACCGTACGCTCCAAATCAG-3’

Runs of G deoxynucleotides in wt are in bold, as are the corresponding runs in the variant; underlined deoxynucleotides are variations from wt.

**Table 1:** Oligodeoxynucleotide (ODN) inserts and phenotypes observed.

		Effect^a^			
		C/A (G4 template)		Attenuation (G4 nascent)	
	Plasmid insert	GW	GS	GW	GS
G4-1-a	5’-GATCCAATAGGAGA**GGGG**A**GGGG**AA**GGGG**A**GGGG**AAAAGGTAAG-3’	+++	+	+++	+++
G4-1-b	3’-GTTATCCTCTCCCCTCCCCTTCCCCTCCCCTTTTCCATTCTTAA-5’				
G4-1-r-a	5’-GATCCTTACCTTTTCCCCTCCCCTTCCCCTCCCCTCTCCTATTG-3’	+++	-/+	-^b^	+++
G4-1-r-b	3’-GAATGGAAAA**GGGG**A**GGGG**AA**GGGG**A**GGGG**AGAGGATAACTTAA-5’				
G4-1-v1-a	5’-GATCCAATAGGAGA**GCGG**A**GCGG**AA**GCGG**A**GGGG**AAAAGGTAAG-3’	-	-	-	-
G4-1-v1-b	3’-GTTATCCTCTCGCCTCGCCTTCGCCTCCCCTTTTCCATTCTTAA-5’				
G4-1-v2-a	5’-GATCCAATAGGAGA**GCGG**A**GGGG**AA**GCGG**A**GCGG**AAAAGGTAAG-3’	-	-	-	-
G4-1-v2-b	3’-GTTATCCTCTCGCCTCCCCTTCGCCTCGCCTTTTCCATTCTTAA-5’				
G4-1-v3-a	5’-GATCCAATAGGAGA**GGGGACGGG**AA**GGGG**A**GGGG**AAAAGGTAAG-3’	-	-	-	-
G4-1-v3-b	3’-GTTATCCTCTCCCCTGCCCTTCCCCTCCCCTTTTCCATTCTTAA-5’				
G4-1-v4-a	5’-GATCCAATAGGAGA**GGGG**A**GGGG**AA**GGGG**A**GGGC**AAAAGGTAAG-3’	-	-	-	-/+
G4-1-v4-b	3’-GTTATCCTCTCCCCTCCCCTTCCCCTCCCGTTTTCCATTCTTAA-5’				
G4-1-v5-a	5’-GATCCAATAGGAGA**CGGG**A**GGGG**AA**GGGG**A**GGGG**AAAAGGTAAG-3’	+	ND	++	ND
G4-1-v5-b	3’-GTTATCCTCTGCCCTCCCCTTCCCCTCCCCTTTTCCATTCTTAA-5’				
G4-1-d-a	5’-GATCCAATAGGAGA**GGGG**A**GGGG** A**GGGG**A**GGGG**AAAAGGTAAG-3’	-	-	-	+++
G4-1-d-b	3’-GTTATCCTCTCCCCTCCCC TCCCCTCCCCTTTTCCATTCTTAA-5’				
G4-1-i-a	5’-GATCCAATAGGAGA**GGGG**A**GGGG**AAA**GGGG**A**GGGG**AAAAGGTAAG-3’	-	ND	+++	ND
G4-1-i-b	3’-GTTATCCTCTCCCCTCCCCTTTCCCCTCCCCTTTTCCATTCTTAA-5’				
G4-1-v6-a	5’-GATCCAATAGGAGA**GGGG**A**GGGG**TT**GGGG**A**GGGG**AAAAGGTAAG-3’	-	ND	+++	ND
G4-1-v6-b	3’-GTTATCCTCTCCCCTCCCCAACCCCTCCCCTTTTCCATTCTTAA-5’				
G4-1-v7-a	5’-GATCCAATAGGAGA**GGGG**A**GGGG**CC**GGGG**A**GGGG**AAAAGGTAAG-3’	-	ND	++	ND
G4-1-v7-b	3’-GTTATCCTCTCCCCTCCCCGGCCCCTCCCCTTTTCCATTCTTAA-5’				
G4-1-v8-a	5’-GATCCAATAGGAGA**GGGG**A**GGGG**TA**GGGG**A**GGGG**AAAAGGTAAG-3’	+	ND	+++	ND
G4-1-v8-b	3’-GTTATCCTCTCCCCTCCCCATCCCCTCCCCTTTTCCATTCTTAA-5’				
G4-1-v9-a	5’-GATCCAATAGGAGA**GGGG**A**GGGG**AT**GGGG**A**GGGG**AAAAGGTAAG-3’	+	ND	+++	ND
G4-1-v9-b	3’-GTTATCCTCTCCCCTCCCCTACCCCTCCCCTTTTCCATTCTTAA				
G4-1-v10-a	5’-GATCCAATAGGAGA**GGGG**T**GGGG**AA**GGGG**A**GGGG**AAAAGGTAAG-3’	+++	ND	+++	ND
G4-1-v10-b	3’-GTTATCCTCTCCCCACCCCTTCCCCTCCCCTTTTCCATTCTTAA-5’				
G4-1-v11-a	5’-GATCCAATAGGAGA**GGGG**A**GGGG**AA**GGGG**T**GGGG**AAAAGGTAAG-3’	-	ND	+++	ND
G4-1-v11-b	3’-GTTATCCTCTCCCCTCCCCTTCCCCACCCCTTTTCCATTCTTAA-5’				
G4-1-v12-a	5’-GATCCAATAGGAGA**GGGG**T**GGGG**AA**GGGG**T**GGGG**AAAAGGTAAG-3’	-	ND	+++	ND
G4-1-v12-b	3’-GTTATCCTCTCCCCACCCCTTCCCCACCCCTTTTCCATTCTTAA-5’				
G4-1-v13-a	5’-GATCCAATAGGAGT**GGGG**A**GGGG**AA**GGGG**A**GGGG**AAAAGGTAAG-3’	+++	ND	+++	ND
G4-1-v13-b	3’-GTTATCCTCACCCCTCCCCTTCCCCTCCCCTTTTCCATTCTTAA-5’				
G4-1-v14-a	5’-GATCCAATAGGAGA**GGGG**A**GGGG**AA**GGGG**A**GGGG**TAAAGGTAAG-3’	+++	ND	+++	ND
G4-1-v14-b	3’-GTTATCCTCTCCCCTCCCCTTCCCCTCCCCATTTCCATTCTTAA-5’				
G4-1-v15-a	5’-GATCCAATAGGACA**GGGG**A**GGGG**AA**GGGG**A**GGGG**AAAAGGTAAG-3’	+++	ND	++	ND
G4-1-v15-b	3’-GTTATCCTGTCCCCTCCCCTTCCCCTCCCCTTTTCCATTCTTAA-5’				
G4-1-v16-a	5’-GATCCAATAGGAGA**GGGG**A**GGTG**AA**GGGG**A**GGGG**AAAAGGTAAG-3’	-	ND	+	ND
G4-1-v16-b	3’-GTTATCCTCTCCCCTCCACTTCCCCTCCCCTTTTCCATTCTTAA-5’				
G4-1-v17-a	5’-GATCCAATAGGAGA**GGGG**A**GGGT**AA**GGGG**A**GGGG**AAAAGGTAAG-3’	-	ND	++	ND
G4-1-v17-b	3’-GTTATCCTCTCCCCTCCCATTCCCCTCCCCTTTTCCATTCTTAA-5’				

Runs of G deoxynucleotides in wt are in bold, as are the corresponding runs in the variant; underlined deoxynucleotides are variations from wt. Sequencing data were acquired from GENEWIZ (GW) and GeneScript (GS).

^a^Scoring systems as follows:—none, + weak, ++ intermediate, +++ strong, ND not determined.

^b^A variant form of pG4-1-r (akin to pG4-1-v1 for pG4-1) was not generated for direct comparison. GENEWIZ data appeared to show no effect (n = 1), while GenScript data showed clear attenuation (n = 3).

### PCR amplification

Plasmids pG4-1 and pG4-1-v1 (5 ηg each) served as templates for PCR amplification (in duplicate, 25 μl per reaction mixture) using 1X buffers supplied by vendors and DNA primers M13F (5’-TGTAAAACGACGGCCAGT-3’) and M13R (5’-CAGGAAACAGCTATGAC-3’) at 0.5 mM each. For high fidelity PCR, deoxynucleoside triphosphates were included at 0.25 mM each, Mg^2+^ at 2 mM, and Pfu Ultra II HS Fusion DNA polymerase (Agilent) at 1/50 reaction volume. For Taq-based PCR amplification, deoxynucleoside triphosphates were included at 0.5 mM each, Mg^2+^ at 2.5 mM, and GoTaq DNA polymerase (Promega) at 1/200 reaction volume (0.625 units). PCR products were purified using the QIAquick PCR Purification Kit (Qiagen).

### DNA sequencing

Plasmids were purified from *E*. *coli* DH5α using the QIAprep Spin Miniprep Kit (Qiagen) and dissolved in 10 mM Tris-HCl, pH 8.5 at 80 ng/ul. Samples of 800 ng were submitted to either GENEWIZ or GenScript for Sanger DNA sequencing with dye-labelled dideoxynucleoside triphosphates [35]. M13-based sequencing primers were provided by the vendors: GENEWIZ: M13F, M13F(-47), and M13R; GenScript: M13F and M13R. Most inserts were sequenced at least twice, either using the same construct or independent isolates. The following two 100 nucleotide ODNs (IDT) were sequenced using primer G4-1F, 5’-TGGGTACCGGGCCCC-3’: G4-1-ssDNA, 5’-GGATCCAATAGGAGA**GGGG**A**GGGG**AA**GGGG**A**GGGG**AAAAGGTAAGAATTCGATATCAAGCTTATCGATACCGTCGACCTCGAGGGGGGGC CCGGTACCCA-3’; G4-1-v4 ssDNA, 5’-GGATCCAATAGGAGA**GGGG**A**GGGG**AA**GGGG**A**GGGC**AAAAGGTA AGAATTCGATATCAAGCTTATCGATACCGTCGACCTCGAGGGGGGGCCCGGTACCCA-3’. Runs of G deoxynucleotides in the wt are in bold, as are the corresponding runs in the variant with the altered deoxynucleotide underlined.

### Native gel electrophoresis

ODNs were prepared at 10 μM in 25 mM Tris-Cl, pH7.5 and heated at 94–95°C for 5 min. The samples were then allowed to slowly cool to ~room temperature and then placed on ice. Either 5 μl water or 5 μl 0.5 M KCl or LiCl were added to 20 μl aliquots and the samples were incubated at 23°C for 40 hours. Loading buffer was added and the samples were applied to 15% 19:1 acrylamide/bis-acrylamide gels containing 1X TBE and 20 mM KCl or LiCl. Electrophoresis was conducted at 4°C with 1X TBE / 20 mM KCl or LiCl running buffer and at constant voltage. Bands were visualized by staining with 0.03% methylene blue.

### Fluorescence-based detection of G4 structures

ODNs were prepared as for gel electrophoresis. In this case, either 10 μl water or 10 μl 0.5M KCl were added to 40 μl aliquots and the samples were incubated at 23°C for 48 hours. Triplicate aliquots (5 μl) were added to 200 μl 25 mM Tris-Cl / 1 μM NMM in a 96 well plate and the samples were incubated in the dark at room temperature for 15 min. Fluorescence intensity (excitation 393 nm, emission 610 nm) was measured with a Synergy H1. Control ODNs included the following: CMYC, 5’-TGGCGACGGCAGCGAGGCGGGTGGGTAGGGTGGG-3’; (TTA)_3_, 5’- TGGCGACGGCAGCGAGGCGGGTTAGGGTTAGGGTTAGGG-3’; and (TTA)_3_-G, 5’- TGGCGACGGCAGCGAGGCGGGTTAGGGTTAGGGTTAG-3’ (see ref. [40]).

### Peak height analysis

For peak height analysis, data were extracted from .ab1 files using a tool available online on the Thermo- Fisher Connect platform. Peak height values were normalized by dividing the peak height by the mean peak height of the pre-G4 region. In most cases, this denominator was derived from 20 values. However, two variations were in the upstream region; in these cases, the denominator was derived from 19 values (G4-1-v13) or 18 values (G4-1-v15).

### G4 motif search

Localization of the G4-1 sequence in the human genome was conducted using the Blast-like Alignment Tool (BLAT) to interrogate the human genome GRCh38/hg38 assembly [37].

## Results and discussion

### A natural G4 sequence from budding yeast

To generate a reagent to be used for a genetic screen, plasmid pG4-1 was constructed containing a DNA sequence from the *S*. *cerevisiae* genome (chr. IV) that is known to form a G4 structure *in vitro* and that was identified as an evolutionarily conserved element [34]. In this prior study, circular dichroism analysis revealed that the sequence 5’-G_4_A_1_G_4_A_2_G_4_A_1_G_3_-3’ forms a parallel G4 structure defined by the four strands having the same 5’-3’orientation. The pG4-1 plasmid contains this sequence and endogenous flanking sequences from its *S*. *cerevisiae* locus (38 base pairs total including the 19 base pair G4 region). In this natural sequence, an additional G is present on the 3’ end of the G4 sequence cited above, leading to the G4 sequence 5’-G_4_A_1_G_4_A_2_G_4_A_1_G_4_-3’. Nuclear magnetic resonance analysis on a related DNA molecule found in *Tetrahymena* telomeres, (G_4_T_2_)_4_, revealed a mixed parallel and anti-parallel conformation [38]. In that study, it was found that only three stacked G-tetrads form intramolecularly despite the possibility of a fourth. Thus, G deoxynucleotides can contribute to the loop regions in these types of sequences. Other work using spectroscopic and electrophoretic techniques has indicated that (T_2_G_4_)_4_ can form at least two structures [39]. It is clear that solution conditions, including the nature of the cation used [3], impact the G4 structure.

### A novel sequencing anomaly arising from the G4-1 template and analysis of variants in the G-rich region

**Fig 1:**
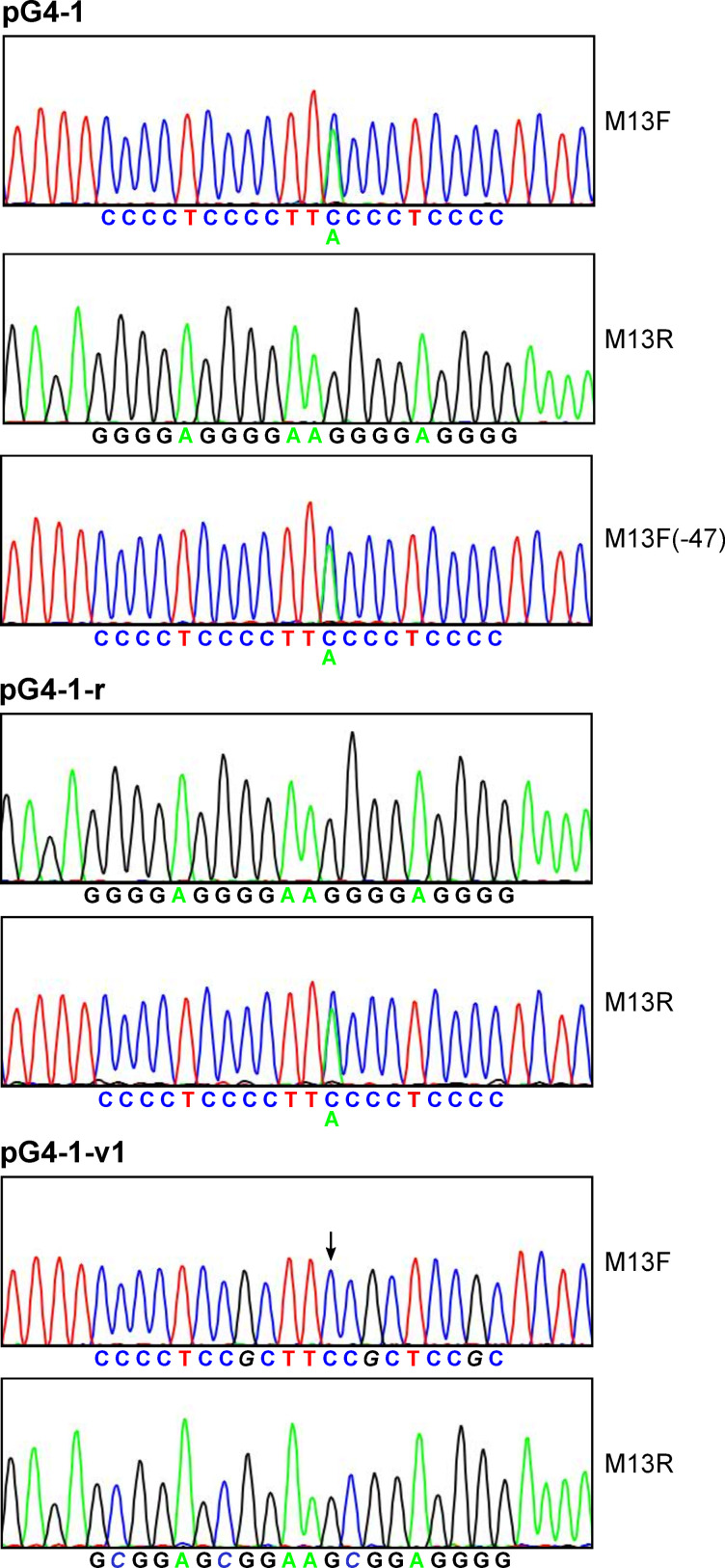
G4-1 template causes coincident C and A signals within the G4 sequence. DNA sequence tracings of pG4-1, pG4-1-r, and pG4-1-v1 are shown, with primers employed as indicated. Sequences are provided in a color-coded fashion below the tracings, with variations in italics. The arrow in the bottom panel (pG4-1-v1) indicates the position corresponding to the wild-type effect.

To confirm that the pG4-1 construct was correct, the insert DNA sequence was determined by Sanger dye-terminator sequencing using a commercial vendor (GENEWIZ). Trace results are shown in Fig 1 (top). As can be seen, the sequence from the M13F primer gave rise to coincident C and A peaks at an internal position in the putative G4 structure. The sequence from the M13R primer did not show a similar anomaly in this region, indicating that the template DNA was not a mixed population. Another sequencing analysis conducted with a proprietary protocol specially designed for GC-rich regions gave the same M13F-specific result (S1 Fig). The primer position did not have an influence on the effect as the appearance of an A residue was observed using the M13F(-47) primer (Fig 1, top). In addition, a construct with the insert in the opposite orientation (pG4-1-r) was generated. In this case, sequencing from the M13R primer provided the identical anomaly, as would be expected if the effect were dependent on the G4 structure and not the primer or orientation of plasmid sequences (Fig 1, middle). A control construct intended for the screen was generated in which one G in each of the first three G runs (from 5’ to 3’) was replaced with a C (pG4-1-v1; see Table 1 for the sequence of this variant and others referenced in the text, and a summary of all phenotypes). In theory, this sequence would not form an intramolecular G4 structure like that of the wild type (wt) sequence but it contains the same GC content. When sequenced, the coincident C and A peaks were not observed (Fig 1, bottom). It is noted that the normal DNA sequencing protocol included K+, confirmed by representatives of GENEWIZ. Therefore, sequencing conditions are suitable for G4 formation.

The same constructs were sequenced by a different vendor, GenScript, with differing results. In this case, the anomaly observed with pG4-1 was seen in 3 of 4 sequencing runs but was subtle (S2A Fig). The small effect was not observed with pG4-1-v1, consistent with the idea that the G4 DNA was responsible. According to representatives of GenScript, their normal DNA sequencing protocol also includes K+, suitable for G4 formation. Clearly, however, there are specific conditions required for a robust effect.

**Fig 2:**
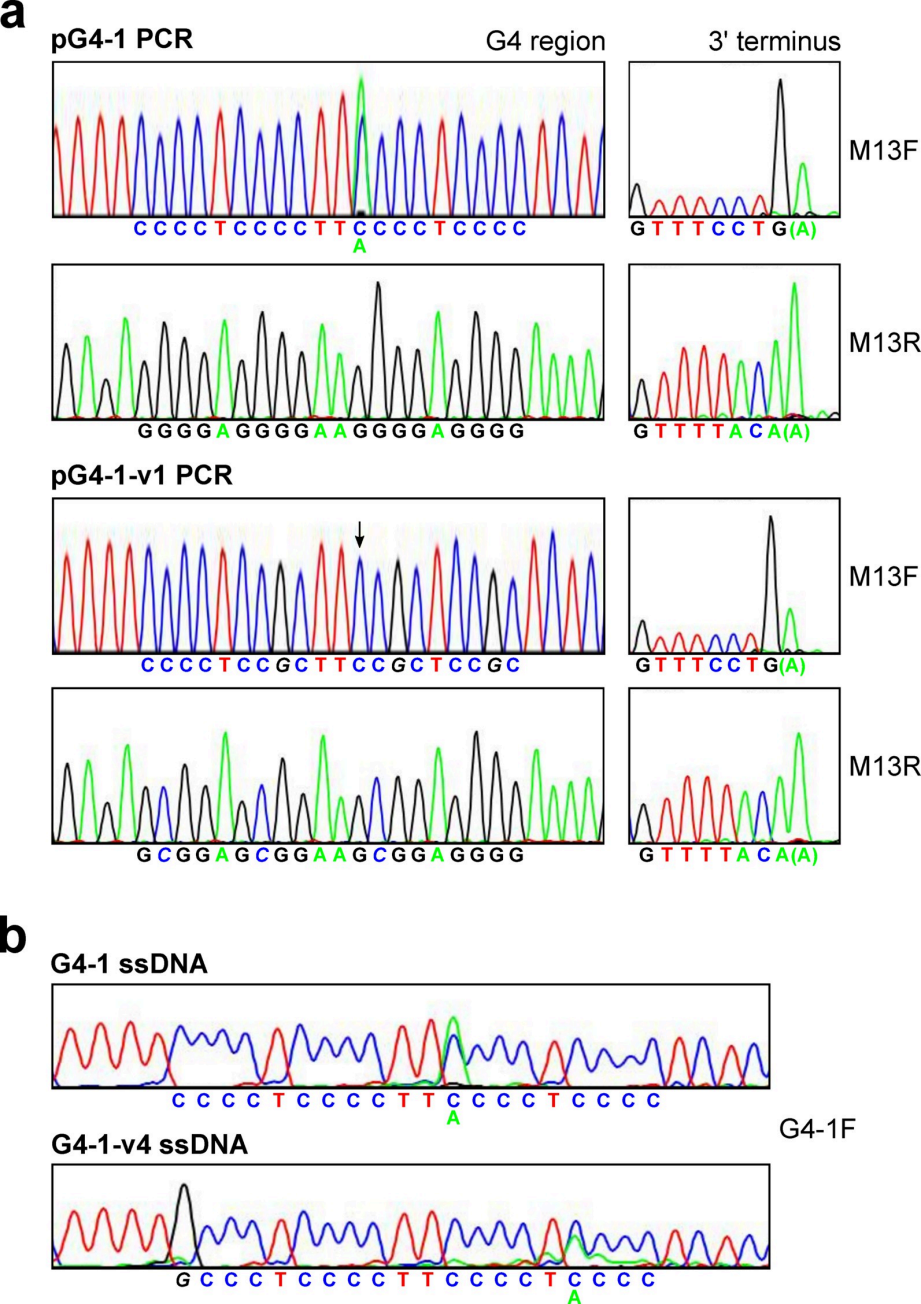
PCR product and ODN sequencing of the G4-1 region exhibit the C/A anomaly. (*a*) Plasmids pG4-1 and pG4-1-v1 were amplified by PCR with Pfu and the resulting products were sequenced with the indicated primers. The left panels include the G4-rich strands and complementary C-rich strands for wt and variant, as indicated. The right panels include the 3’-termini of the products, with expected final sequence indicated below each tracing and the extra A in parentheses. Note that the peaks from the sequencing reactions using the M13F appear diminished because of the large penultimate G peaks in the 3’ termini. For clarity, the tracings of the G4 regions for these two runs were scaled up vertically. The arrow in the G4-1-v1 PCR panel indicates the position corresponding to the wild-type effect. (*b*) ODNs (100 bp of region) containing either G4-1 or G4-1-4v were sequenced using the G4-1F primer complementary to the 3’ends.

The inserts of pG4-1 and pG4-1-v1 were amplified by polymerase chain reaction (PCR) using a high fidelity Pfu polymerase. Sequencing (GENEWIZ) of the PCR products (254 bp) revealed the same coincident C and A peaks in the G4-1 sample as seen with intact plasmid DNA, and not in the G4-1-v1 sample (Fig 2A). In each case, the 3’-terminus of the sequenced product contained an additional A, as would be expected to result from the terminal transferase activity of a Taq-like DNA polymerase [40]. Because the initial PCR was conducted with Pfu, the terminal A dideoxynucleotide in each reaction was likely a consequence of the polymerase used in sequencing. Sequencing of Taq-amplified PCR products by GenScript showed the subtle C/A anomaly with G4-1 but not with G4-1-v4 (S2B Fig).

An additional experiment was conducted in which a 100 base oligodeoxynucleotide (ODN) containing G4-1 and neighboring residues (see *Materials and methods*) was sequenced. In this ssDNA context, the C/A phenomenon was observed once again. Interestingly, a variant in which the 5’ G of the G4-1 sequence was changed to a C (G4-1-v4) led to a change in the position of the coincident C and A peaks within the G4 sequence with additional neighboring coincident A peaks of lessening intensity (Fig 2B). The same results were observed with GenScript sequencing, although the aberrant A signal was stronger than that of plasmid pG4-1 and the coincident A and C peaks in G4-1-v4 were pronounced (S2C Fig).

Constructs with various G to C changes in the G4-1 sequence were prepared and evaluated once again by sequencing (pG4-1-v2 through -v5). While pG4-1-v5 gave rise to a small A peak, consistent over two sequencing reactions with different plasmid isolates, the other variants were devoid of the anomalous A peak (S3 Fig). Included among these was the G4-1-v4 sequence described above (see Fig 2B), indicating a contrast in result depending on the nature of the template.

### Structural examination of G4-1 and variants

**Fig 3:**
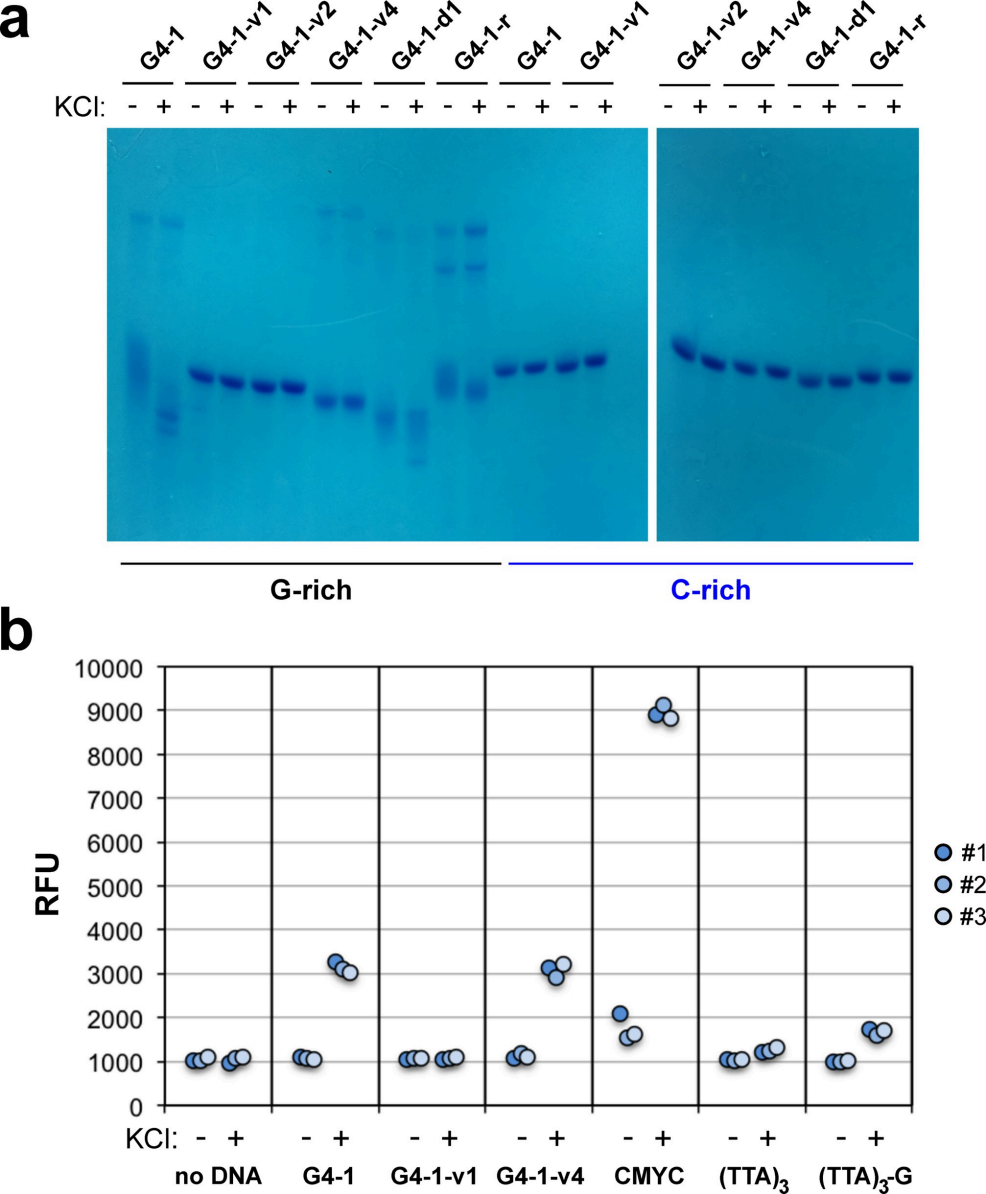
Evidence that the G4-1 sequence forms G4 structures. (*a*) ODNs used to generate pG4-1 and indicated variants were incubated in the absence or presence of 100 mM KCl and subjected to native gel electrophoresis. (*b*) Indicated ODNs were incubated with or without KCl as above and then analyzed for parallel G4 formation using the fluorescent G4 ligand NMM. (Note that the “No DNA” samples for #1 were generated at the time of assay and were not incubated for 48 hrs as in the other two repeats.) Control ODNs included CMYC, (TTA)_3_, and (TTA)_3_-G [43].

Sequences G4-1-v3 through -v5 should be able to form G4 structures because they have single G to C changes in the G4 sequence. To determine whether G4-1 and certain variants could form G4 structures, native gel electrophoresis and fluorescent probe analyses were conducted on ODNs that were used to generate certain constructs. As shown in Fig 3A, the G-rich G4-1 ssDNA showed bands with increased mobility after preliminary incubation with 100 mM KCl, suggesting the generation of intramolecular G4 structures [41]. There were also bands with greatly decreased mobility largely independent of preliminary KCl incubation (note, however, that the gel electrophoresis buffer contained 20 mM KCl). It is possible that these species are intermolecular G4 structures. While the G-rich G4-1-v4 did not show evidence of intramolecular G4 DNA formation in this type of experiment, potential intermolecular structures were observed as with G4-1. By contrast, G-rich G4-1-v1 ssDNA revealed only a single band with or without KCl incubation, as did all of the C-rich ODNs. The same pattern was not seen with LiCl replacing KCl, as lithium ions are not expected to support formation of G4 structures [42] (S4 Fig).

The second type of experiment employed the fluorescent probe N-methyl mesoporphyrin IX (NMM), which targets parallel G4 DNA structures [43–45]. The G-rich G4-1 and G4-1-v4 ODNs exhibited a 3-fold increase in fluorescence over background after incubation with 100 mM KCl, whereas G4-1-v1 did not (Fig 3B). Included were control ODNs CMYC, which forms a parallel G4 DNA structure, (TTA)_3_, which forms an antiparallel G4 structure, and (TTA)_3_-G, which only has three runs of Gs and is not expected to form a G4 structure [43]. CMYC and (TTA)_3_ behaved as expected in response to NMM, whereas (TTA)_3_-G showed a slight increase in fluorescence dependent on KCl incubation. Despite this slight anomaly, the data provide evidence that G4-1 and G4-1-v4 G-rich ssDNAs can form parallel G4 structures. There appears to be a particular G4 structure formed by the G4-1 sequence that can influence DNA polymerase in the context of dye-terminator sequencing, and it is possible that alternative G4 structures are formed in certain variants that are not particularly impactful.

### Variations in the G4-1 loop and neighboring regions

Additional variants were constructed that included changes in the loop regions of the G4 sequence (S5 Fig). The central loop was modified by deleting an A residue (G4-1-d1) or inserting an additional A (pG4-1-i1). Both changes abolished the appearance of coincident C and A peaks. Changing the two As to Ts or Cs (pG4-1-v6 and pG4-1-v7, respectively) also led to a normal sequence profile. In the latter case, the GC content was even higher than that of G4-1, further indicating that something other than DNA polymerase encountering a G-rich-template was responsible for the effect. Changing one A to T (G4-1-v8 and v9) greatly diminished the appearance of the aberrant A peak. Experiments with the other two loop regions provided mixed results. Substituting A with T in the first loop (as read 5’ to 3’; G4-1-v10) led to a sequence profile with coincident C and A peaks. However, making the same change to the third loop (G4-1-v11) abolished the effect, as did making the same substitutions in both loops (G4-1-v12) (S6 Fig). These results indicate that the composition of the first two loop regions that the polymerase encounters, preceding the location of the coincident peaks, has an impact on the observed phenotype within the context of these particular changes.

Residues immediately flanking the G4 sequence were also analyzed. Altering the 5’ A or 3’ A to a T (pG4-1-v13 and -v14, respectively) did not diminish the generation of coincident peaks; substituting the G residue two nucleotides upstream of the G4 sequence with a C (pG4-1-v15) likewise had no effect (S6 Fig). Thus, the residues found within the proposed G4 sequence rather than neighboring residues are responsible for the phenomenon.

### Sequence analysis of mixed populations

**Fig 4:**
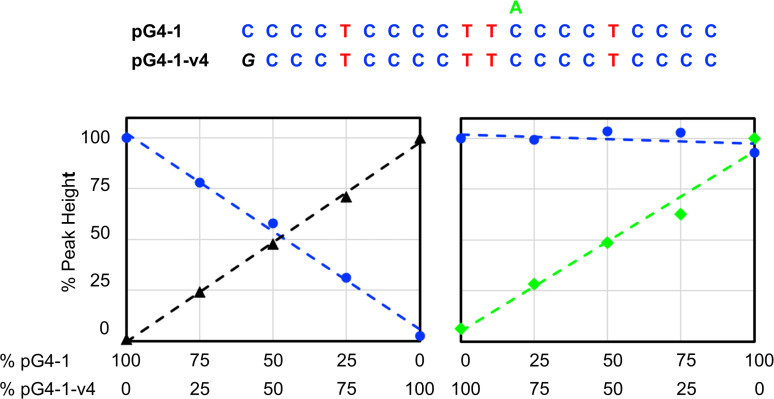
DNA sequence peak heights from mixtures of wt G4-1 and a variant reveal a difference in pattern. Indicated ratios of pG4-1 and pG4-1-v4 were mixed and sequenced. Peak heights (normalized, see *Materials and methods*) for wt C residues and the variant G and the anomalous A residues were calculated as percentages of the maximum peak height observed for that residue.

Close consideration of the coincident C and A peaks in G4-1 relative to a variant that does not exhibit this effect suggests that the appearance of the A peak does not diminish the C peak height to a significant extent. If there were misincorporation or a terminal transferase-mediated event with relatively high frequency, as suggested by the A peak height, the C peak height should be affected. To test this, an experiment was conducted in which pG4-1 and pG4-1-v4 were mixed in different proportions and sequenced. As expected, the peak heights of the residue that differed in the two constructs were directly proportional to the percentages of constructs in the mix (Fig 4). Thus, a 50/50 mixture of pG4-1 and pG4-1-v4 gave peaks one-half the height of those observed with either plasmid alone. By contrast, the appearance of the A peak (from pG4-1) had very little effect on the C peak height. These data suggest that misincorporation was not likely the cause of the effect.

One possible explanation for the mixing experiment result is that a “compression” occurred during the DNA sequencing reaction. In this scenario, an extra A would be added to the sequence leading to a terminated DNA molecule that has abnormal chromatographic properties and as a result does not resolve properly. To examine this possibility, two variations in the G4 sequence were made in which a T replaced a G in the G4 sequence on either side of the G that is the effected site (pG4-1-v16 and -v17). In both cases, the sequence was normal (S7 Fig), suggesting that an abnormality in DNA migration was not responsible.

The DNA sequence profiles of pG4-1-v16 and -v17 showed a large difference in the A peak heights resulting from the T substitutions, with the former being smaller in relation to neighboring A peaks, and the latter being larger. Therefore, sequence context in this region has a noticeable effect on the incorporation of the dye terminator. Given the results of the mixing experiment, it is possible that dye-labeled ddATP is incorporated more efficiently than dATP, leading to an exaggerated peak without much effect on the C peak. This possibility would mean that the incorporation of an A was not as frequent as the peak height suggests.

### Theoretical G4-1 structures

**Fig 5:**
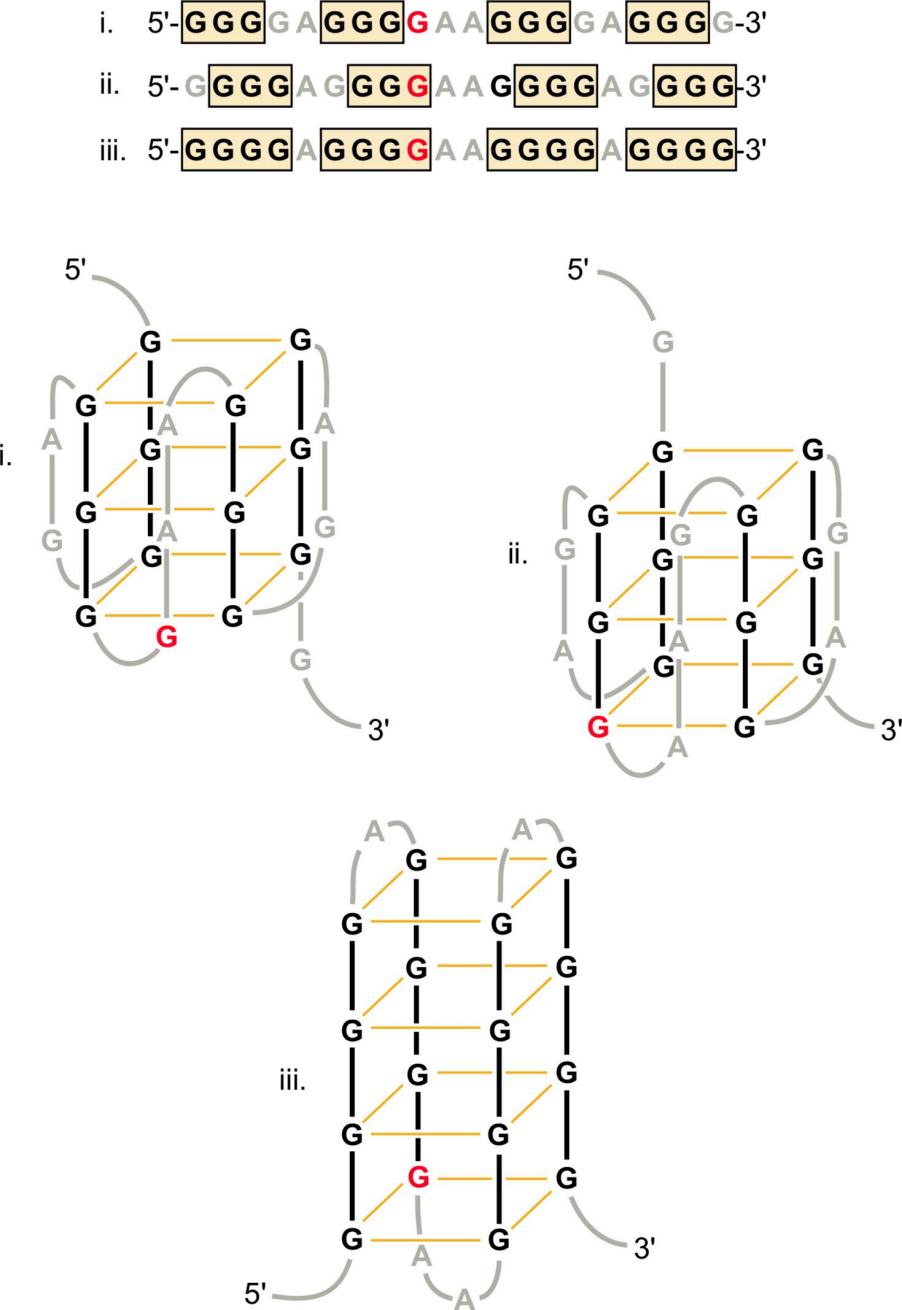
Theoretical structures formed by the G4-1 sequence. Models *i* and *ii* are based on the report that the G4-1 sequence, but lacking the 3’ G, forms a parallel structure [34]. Model *iii* is a hypothetical antiparallel structure involving four stacked planes of Hoogsteen-paired G deoxynucleotides rather than three. G deoxynucleotides and covalent linkages participating directly in the G4 formation (boxed in primary sequences at top) are in black, with Hoogsteen base pairing indicated by the orange lines. Deoxynucleotides and covalent linkages in loop regions are in gray. The G position that leads to coincident C and A peaks is shown in red (loop element in model *i*, G4 element in *ii* and *iii*).

Among possible mechanisms for the observed double peaks is that a component of certain G4 structure mimics blunt-ended duplex as the DNA polymerase proceeds, leading to activation of the terminal transferase activity and addition of a terminal A residue as opposed to the normal C. The PCR reactions described above (see Fig 2A) did not lead to a change of sequence on both strands, as would be expected if a mismatch were occurring at a reasonable frequency. It is recognized, however, that the effect may be limited to certain DNA polymerases or conditions, as evidenced by studies with a different vendor, GenScript, for which this phenomenon was weak and variable (see Table 1). In the theoretical parallel G4 structures shown in Fig 5, an inhibitory element could include a G-hairpin-like structure that remains stable even as the DNA polymerase traverses through the first half of the G4 sequence. Structure *iii* would appear to provide a satisfying template for the proposed termination mechanism, and this structure (along with *ii*) would be prevented in the G4-1-v4 variant containing the single 3’ G to C change. Such an argument can be made for the G4-1-v3 and -v5 variants as well, in these cases eliminating the possibilities of structures *i* and *iii*. It should be emphasized that there are numerous theoretical G quadruplex structures that can be formed through these various sequences. The program QGRS Mapper [46], which includes a prediction algorithm, provides 206 different possible arrangements for the G4 sequence within G4-1, with the structure including all 16 G residues involved in the G4 structure receiving a high G-score of 62 (out of 105). For comparison’s sake, the highest scoring structure of the well-known c*MYC* G4 sequence receives a G-score of 41. The two G4-1 variants discussed above have 125 possibilities, with the highest G-score at 42. The fact that significant C peaks were observed would suggest that there is an equilibrium between a deleterious G4 structure and other G4 structures as well as the canonical DNA structure that does not impede DNA polymerase progression. It should be noted that “internal” arrest of DNA synthesis has been observed with long tracts of TC or GA repeats, in these cases due to formation of triplex structures that structurally prevent further DNA synthesis [47]. It has also been reported previously that Sanger sequencing (using radioactivity as opposed to fluorescence) can be used to reveal G4 structures on the template strand. In this case, however, sequencing termination was reported to occur at the beginning of the G4 structures, as might be expected, and consistent with other *in vitro* studies on DNA polymerases cited above [21]. Thus, the effect in the internal region of the G4 structure is a new observation.

### Inhibition of DNA synthesis by G4 in the nascent strand

**Fig 6:**
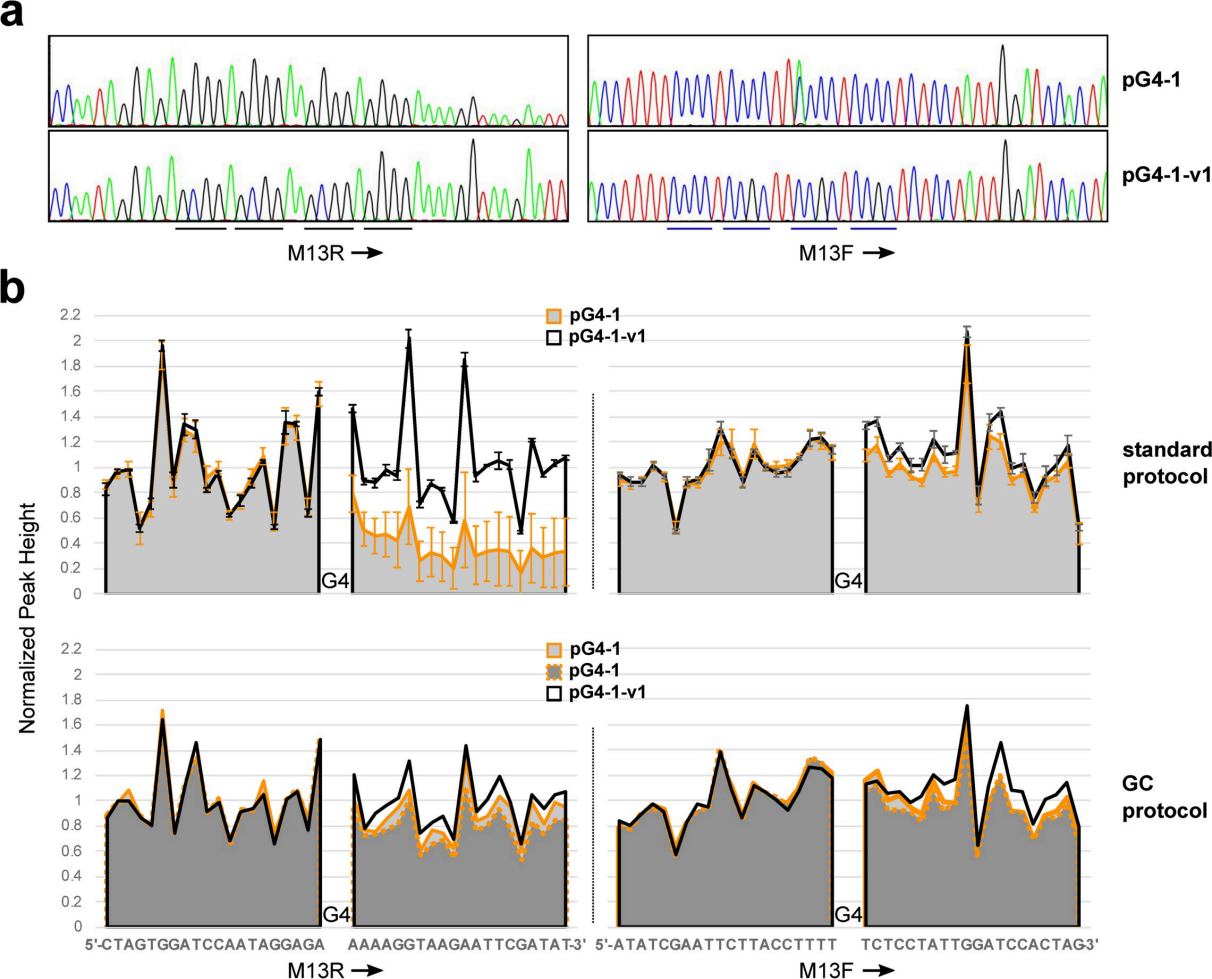
Attenuation of DNA synthesis by nascent strand G4-1. (*a*) Representative DNA sequence tracings (GENEWIZ standard protocol) are shown with plasmids and primers employed as indicated. The lines below the tracings indicate the positions of the G4 repeats (wt or variant) and complementary C-rich sequences. (*b*) The charts show the average normalized peak heights for pG4-1 (10 runs with M13R, 12 runs with M13F including one with M13F(-47)) and pG4-1-v1 (4 runs with M13R and 4 runs with M13F including one with M13F(-47)) before and after the G4 region (wt or variant) upon standard protocol sequencing. Error bars indicate standard deviation. The lower charts show the normalized peak heights for pG4-1 (one run) and pG4-1-v1 (two runs) using the protocol designed for GC rich sequences.

During the course of these studies, it was noted that attenuation of DNA synthesis upon DNA polymerase traversing the C-rich region of G4-1 DNA occurred during GENEWIZ sequencing with some variability. Quantification of peak heights from multiple sequencing runs revealed the degree of inhibition (Fig 6). This decrease in peak height was not observed with pG4-1-v1. Unlike the coincident C/A peak effect, attenuation was abolished when a sequencing protocol for GC rich regions was used (see S1 File for all data and plots). These constructs were also submitted to GenScript for sequencing. In this case, the effect was enhanced and more consistent compared to the GENEWIZ results (S8 Fig). Other variants described above were analyzed for attenuation (GENEWIZ and GenScript). The results are summarized in Table 1. While it is difficult to reach solid conclusions with the variability inherent to this phenomenon, there is a reasonable correlation between the severity of attenuation and the C/A effect in variants that have altered G4 repeats and those with changes outside the G4 region. However, variants with alterations in the loop regions did not correlate as well in that several exhibited strong attenuation but not coincident C and A peaks. Therefore, the attenuation effect may be more general than the C/A effect.

PCR products of wt and variant G4-1 regions (254 bp) were sequenced by both vendors. There was very little effect in all three experiments, and variability between duplicates where a possible effect was observed with G4-1 (see S1 File). It is possible that strong attenuation in this system depends on the nature of the sequencing template.

It has been observed previously *in vitro* that non-canonical i-motif structures formed by a C-rich strand opposite a G4 sequence can inhibit DNA synthesis by Klenow Fragment [24]. However, this inhibition depends on lowering the pH to 6.0. In addition, the inhibition occurs at the beginning of the i-motif sequence. In the case shown here, inhibition began to occur as the DNA polymerase crossed the C-rich sequence. Therefore, it appears that inhibition depends on the formation of a specific structure on the nascent strand.

### An additional natural G4 sequence from budding yeast

**Fig 7:**
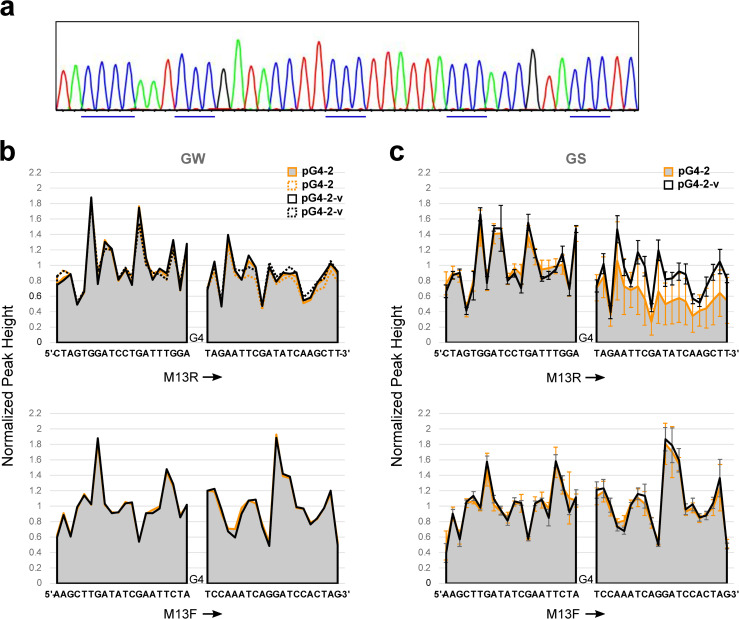
A second G4 sequence can lead to nascent strand-mediated attenuation of DNA synthesis. *(a)* Sequence analysis of pG4-2 using the M13F (-47) primer (GENEWIZ). The runs of Cs are indicated with blue lines. *(b)* Normalized peak heights for DNA sequencing carried out by GENEWIZ (GW) and (*c*) GenScript (GS) are shown for pG4-2 and its mutant derivative, pG4-2-v. Indicated primers were used. For (*b*), the plasmids were sequenced using M13R two times and M13F one time. For (*c*), the plasmids were sequenced four times with M13R, and pG4-2 was sequenced three times and pG4-2-v four times with M13F. Error bars indicate standard deviation.

Another evolutionarily conserved *S*. *cerevisiae* DNA sequence (chr. IX) that is known to form a G4 structure [34] was cloned and interrogated by Sanger sequencing. In the case of G4-2, the loop regions were longer, and the four G runs contained three G deoxynucleotides (although the endogenous flanking sequence added an additional run of four Gs; see *Materials and methods*). Coincident peaks were not observed in the wt or variant sequence (Fig 7A). This result reinforces the idea that G4-1 (as well as certain variants described in this study) has a specific structure leading to this particular effect.

Plasmids pG4-2 and pG4-2m were also analyzed for attenuation, with variable results: in some cases, there was no effect, in other cases inhibition of DNA synthesis was observed as the DNA polymerase synthesized the wt, but not the mutant, G4 sequence (Fig 7B and 7C). Therefore, the effect of the nascent strand, although highly sensitive to specific conditions, may apply to G4 structures other than G4-1, as suggested above.

### G4-1 sequences in the human genome

The effects on DNA replication discovered here could be relevant to human health if they are not restricted to modified Taq-like DNA polymerases and dye-terminator sequencing conditions. A search for the G4-1 sequence in the human genome revealed 76 exact matches spread over 16 of 22 autosomes along with the X and Y chromosomes (S1 Table). Remarkably, 37% (28) of these are found on chromosome 16. If these G4 structures are problematic for DNA replication even a small percentage of the time on this chromosome or others, perhaps in the absence of certain helicase functions, the consequences in terms of genomic stability and disease could be significant.

## Conclusions

Two anomalies in DNA synthesis have been observed upon dye-terminator Sanger DNA sequencing of G4 sequences. In the first, a simple G4 sequence with small loops leads to what appears to be premature termination in the middle of the G4 sequence. There was a vendor-dependence for this observation, indicating that conditions and/or the nature of the DNA polymerases used dictated outcomes. The second effect results from prevention of DNA synthesis caused by a G4 sequence on the nascent strand. In this case, more consistency was observed between the vendors but did show some variability. Mechanistic insights will require further studies and will reveal whether the phenomena uncovered here are restricted to the specialized DNA polymerases and conditions used in Sanger DNA sequencing or are more generalizable.

## Supporting information

S1 Table Occurrences of the G4-1 sequence in the human genome. (DOCX)

S1 Fig Sequencing protocol for GC-rich regions does not abolish the C/A anomaly. Plasmid pG4-1 was sequenced with indicated primers using a proprietary protocol (GENEWIZ) designed to eliminate difficulties in sequencing GC-rich regions.
(TIF)

S2 Fig Modest effects using a different sequencing vendor. Traces of *(a)* plasmids, (*b*) PCR products, and (*c*) ssDNA templates sequenced by GenScript using the indicated primers are shown. As in Fig 2 the tracings of the G4 regions for the PCR runs were scaled up vertically. The arrows indicate the positions corresponding to the wild-type effect.
(TIF)

S3 Fig Single deoxynucleotide variations in the G4 repeats of plasmid DNA abolish the C/A anomaly. DNA sequence tracings variants, including three with single deoxynucleotide changes, are shown with plasmids and primers employed as indicated. The arrows indicate the positions corresponding to the wild-type effect.
(TIF)

S4 Fig Comparison of potassium and lithium ions in capacity for G4 formation. ODNs used to generate pG4-1 and indicated variants were incubated in the absence or presence of 100 mM KCl or LiCl and subjected to native gel electrophoresis.
(TIF)

S5 Fig Analysis of center loop variants for the C/A anomaly. The central TT sequence was altered to change the length or composition and resulting constructs were sequenced using the M13F primer. The arrows indicate the positions corresponding to the wild-type effect.
(TIF)

S6 Fig Analysis of loop 1 and 3 variants for the C/A anomaly. T to A changes were made in loops 1, 3, or both, and the resulting constructs were sequenced using the M13F primer. The arrows indicate the positions corresponding to the wild-type effect.
(TIF)

S7 Fig Test for compression. Variants were generated so that an A would be incorporated in one of two locations during sequencing (pG4-1-v16 and -v17). Tracings in comparison to pG4-1 are shown.
(TIF)

S8 Fig Attenuation of DNA synthesis by nascent strand G4-1. (*a*) Representative DNA sequence tracings (GenScript standard protocol) are shown for pG4-1 and pG4-1-v1 using the M13R primer. The lines below the tracings indicate the positions of the G4 repeats (wt or variant) The chart below indicates the average normalized peak heights before and after the G4 wt (4 runs) or variant (3 runs) sequence. Error bars indicate standard deviation. (*b*) As (a) but with M13F, including 4 runs for pG4-1 and 3 runs for pG4-1-v1.
(TIF)

S1 File Attenuation data. A compilation of DNA synthesis attenuation data and plots.
(XLSX)

S1 Raw images (PDF)
