# Supplementary material for: Anomalies in dye-terminator DNA sequencing caused by a natural G-quadruplex
Source: PLoS One. 2022 Dec 27;17(12):e0279423. doi: 10.1371/journal.pone.0279423 (PMC9794070; doi:10.1371/journal.pone.0279423)
Supplement: S1 Table — (DOCX) [file pone.0279423.s001.docx]

| CHROMOSOME | STRAND | START | END |
| --- | --- | --- | --- |
| chr02 | + | 146696843 | 146696862 |
| chr04 | - | 78217963 | 78217982 |
| chr04 | - | 1408099 | 1408118 |
| chr05 | + | 157126459 | 157126478 |
| chr06 | - | 139067754 | 139067773 |
| chr06 | + | 53724626 | 53724645 |
| chr07 | - | 100282521 | 100282540 |
| chr07 | - | 75254683 | 75254702 |
| chr07 | + | 128701348 | 128701367 |
| chr07 | + | 89559718 | 89559737 |
| chr07 | + | 68691087 | 68691106 |
| chr08 | - | 85833020 | 85833039 |
| chr08 | + | 108601159 | 108601178 |
| chr09 | - | 133590110 | 133590129 |
| chr09 | - | 130315234 | 130315253 |
| chr09 | - | 41366984 | 41367003 |
| chr09 | - | 37188807 | 37188826 |
| chr09 | - | 34220032 | 34220051 |
| chr09 | + | 40098495 | 40098514 |
| chr09_KI270719v1_random | + | 81503 | 81522 |
| chr10 | - | 1138162 | 1138181 |
| chr10 | + | 97251508 | 97251527 |
| chr13 | - | 89174176 | 89174195 |
| chr13 | + | 98730246 | 98730265 |
| chr13 | + | 43756475 | 43756494 |
| chr14 | + | 34027410 | 34027429 |
| chr16 | - | 28758547 | 28758566 |
| chr16 | - | 29038471 | 29038490 |
| chr16 | - | 22519875 | 22519894 |
| chr16 | - | 16336560 | 16336579 |
| chr16 | - | 17071749 | 17071768 |
| chr16 | - | 14712322 | 14712341 |
| chr16 | - | 14751446 | 14751465 |
| chr16 | - | 14938076 | 14938095 |
| chr16 | - | 7022020 | 7022039 |
| chr16 | + | 69990139 | 69990158 |
| chr16 | + | 29499518 | 29499537 |
| chr16 | + | 30239486 | 30239505 |
| chr16 | + | 28356721 | 28356740 |
| chr16 | + | 28470559 | 28470578 |
| chr16 | + | 21419010 | 21419029 |
| chr16 | + | 21851290 | 21851309 |
| chr16 | + | 18372136 | 18372155 |
| chr16 | + | 15122824 | 15122843 |
| chr16_KI270853v1_alt | - | 1995741 | 1995760 |
| chr16_KI270853v1_alt | - | 285184 | 285203 |
| chr16_KI270853v1_alt | - | 542400 | 542419 |
| chr16_KI270853v1_alt | - | 777399 | 777418 |
| chr16_KI270853v1_alt | + | 731645 | 731664 |
| chr16_KV880768v1_fix | - | 1253725 | 1253744 |
| chr16_KV880768v1_fix | - | 1685723 | 1685742 |
| chr16_KV880768v1_fix | + | 241780 | 241799 |
| chr16_KV880768v1_fix | + | 585147 | 585166 |
| chr16_KZ559113v1_fix | - | 73010 | 73029 |
| chr17 | - | 43361740 | 43361759 |
| chr17 | + | 55431921 | 55431940 |
| chr17 | + | 41101709 | 41101728 |
| chr17_JH159146v1_alt | + | 251396 | 251415 |
| chr19 | - | 41952149 | 41952168 |
| chr19 | + | 41614936 | 41614955 |
| chr19 | + | 18494246 | 18494265 |
| chr19 | + | 14389280 | 14389299 |
| chr19 | + | 2638465 | 2638484 |
| chr19_KI270867v1_alt | - | 67046 | 67065 |
| chr20 | - | 64233300 | 64233319 |
| chr20_KI270870v1_alt | - | 141891 | 141910 |
| chr21 | + | 38678828 | 38678847 |
| chr21 | + | 31588155 | 31588174 |
| chr21 | + | 31614805 | 31614824 |
| chr21 | + | 10111406 | 10111425 |
| chr22 | - | 19154635 | 19154654 |
| chrX | - | 872875 | 872894 |
| chrX | - | 1514886 | 1514905 |
| chrX | + | 140524585 | 140524604 |
| chrY | - | 872875 | 872894 |
| chrY | - | 1514886 | 1514905 |

**Table S1.** Occurrences of the G4-1 sequence in the human genome.
