# Supplementary figures and images for: Anomalies in dye-terminator DNA sequencing caused by a natural G-quadruplex
Source: PLoS One. 2022 Dec 27;17(12):e0279423. doi: 10.1371/journal.pone.0279423 (PMC9794070; doi:10.1371/journal.pone.0279423)

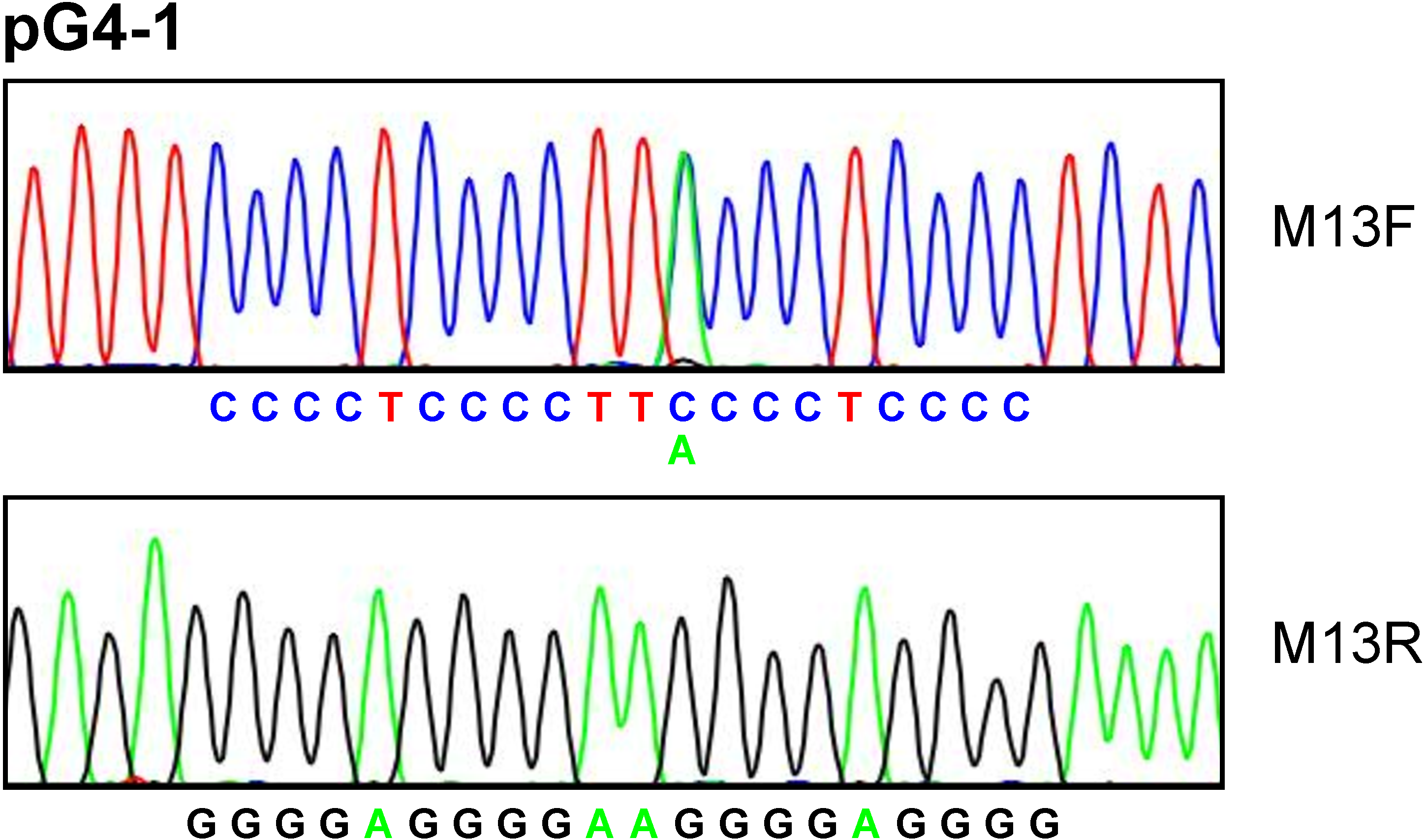

Supplement: S1 Fig — Plasmid pG4-1 was sequenced with indicated primers using a proprietary protocol (GENEWIZ) designed to eliminate difficulties in sequencing GC-rich regions. (TIF) [file pone.0279423.s002.tif]

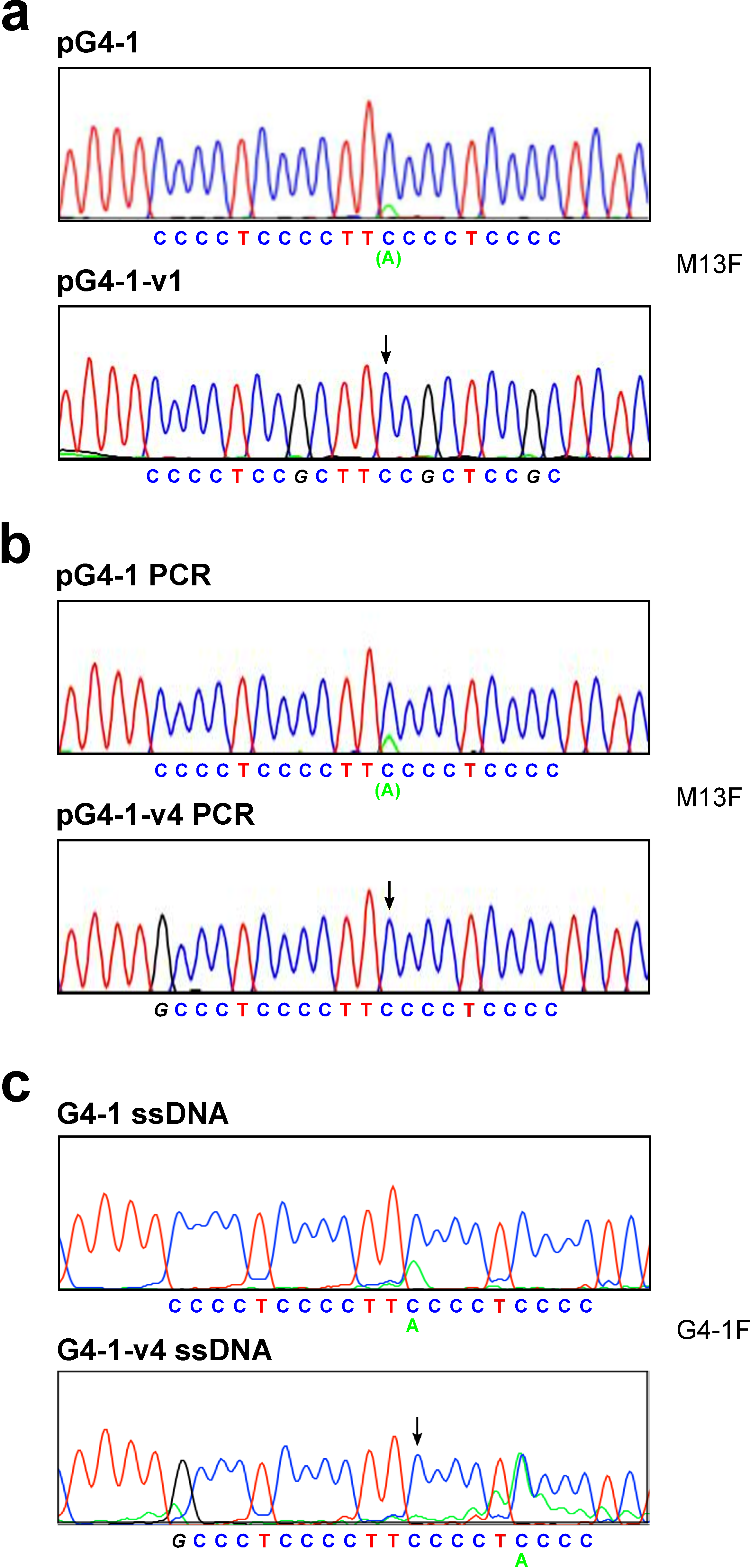

Supplement: S2 Fig — Traces of (a) plasmids, (b) PCR products, and (c) ssDNA templates sequenced by GenScript using the indicated primers are shown. As in Fig 2 the tracings of the G4 regions for the PCR runs were scaled up vertically. The arrows indicate the positions corresponding to the wild-type effect. (TIF) [file pone.0279423.s003.tif]

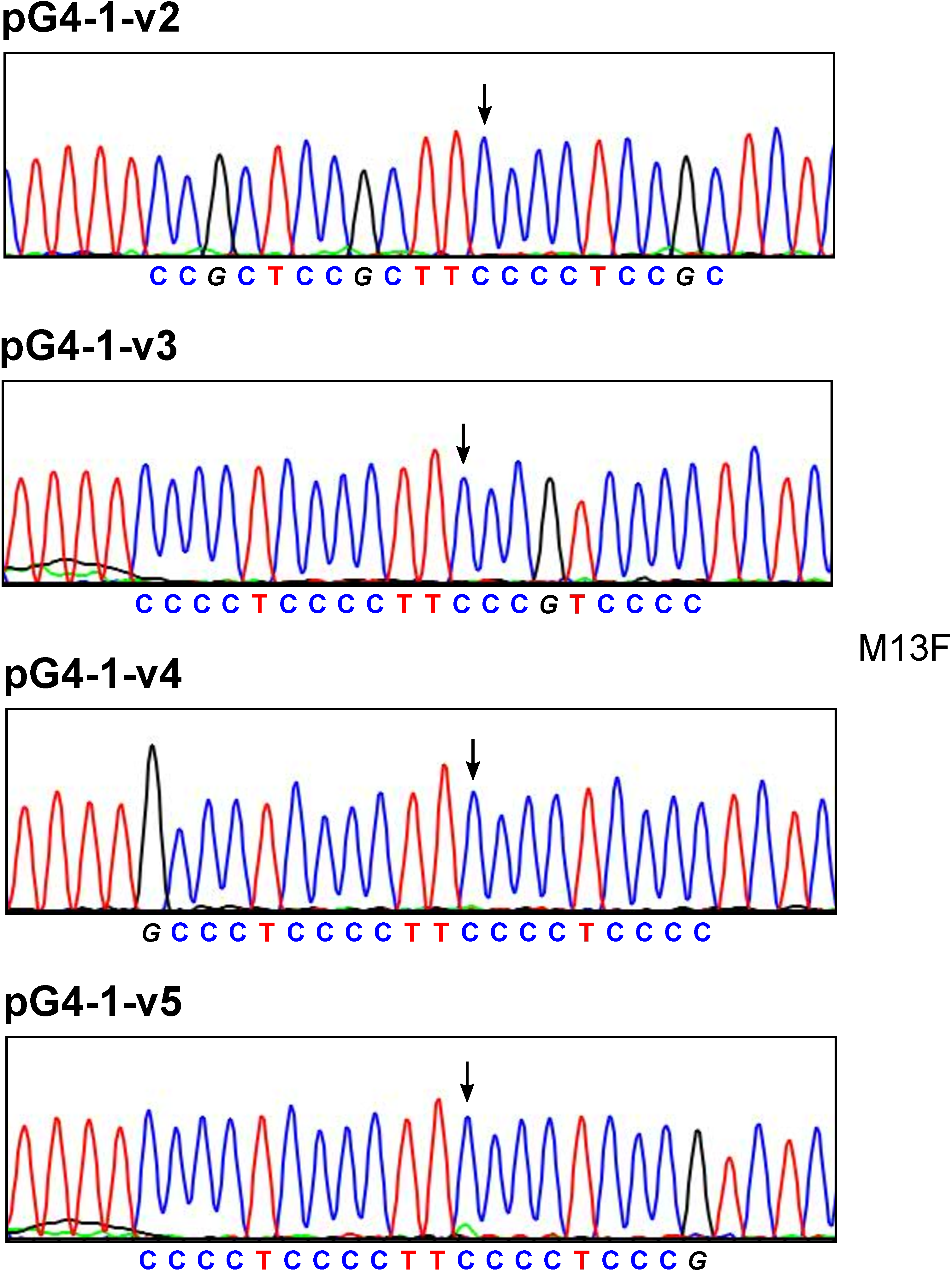

Supplement: S3 Fig — DNA sequence tracings variants, including three with single deoxynucleotide changes, are shown with plasmids and primers employed as indicated. The arrows indicate the positions corresponding to the wild-type effect. (TIF) [file pone.0279423.s004.tif]

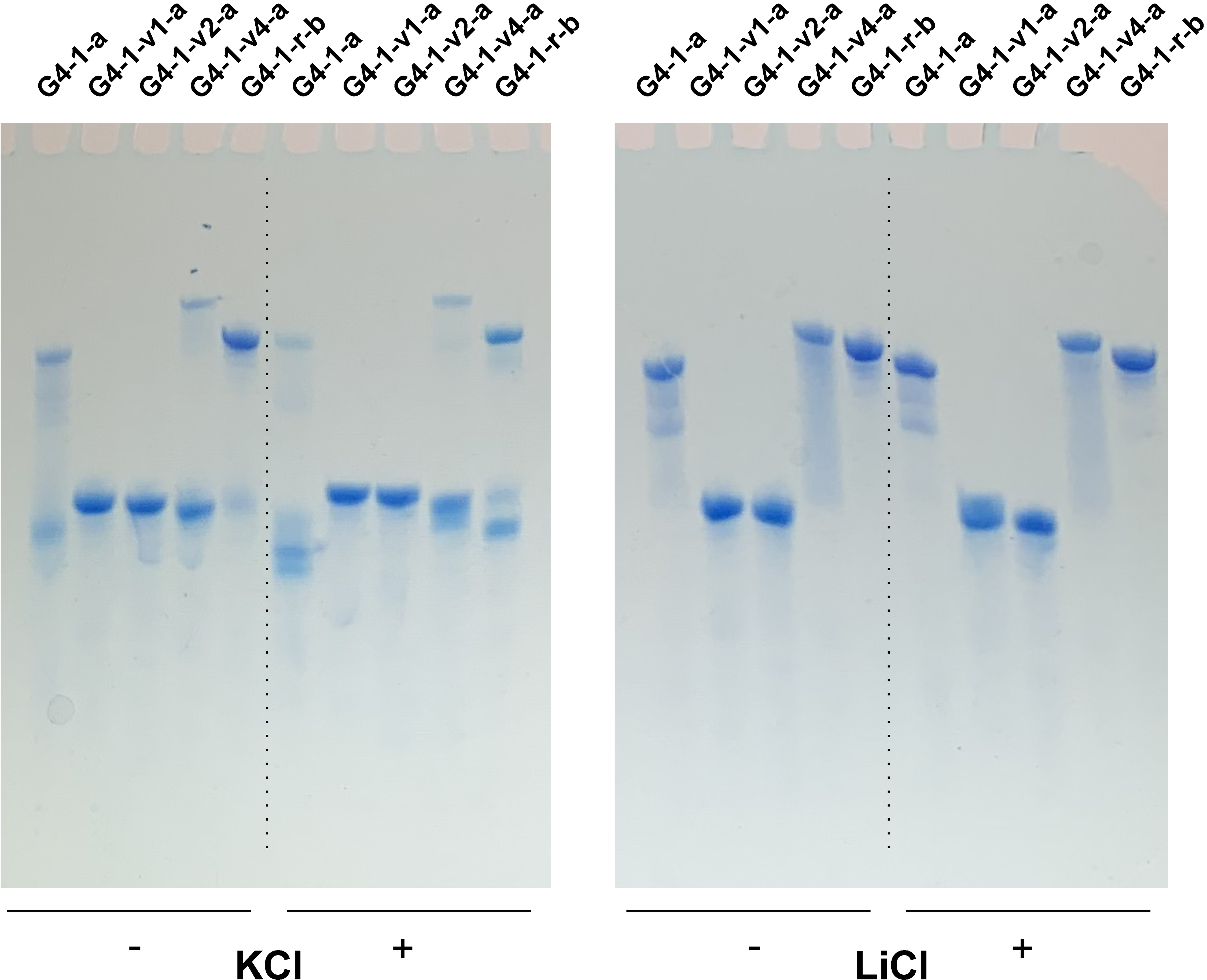

Supplement: S4 Fig — ODNs used to generate pG4-1 and indicated variants were incubated in the absence or presence of 100 mM KCl or LiCl and subjected to native gel electrophoresis. (TIF) [file pone.0279423.s005.tif]

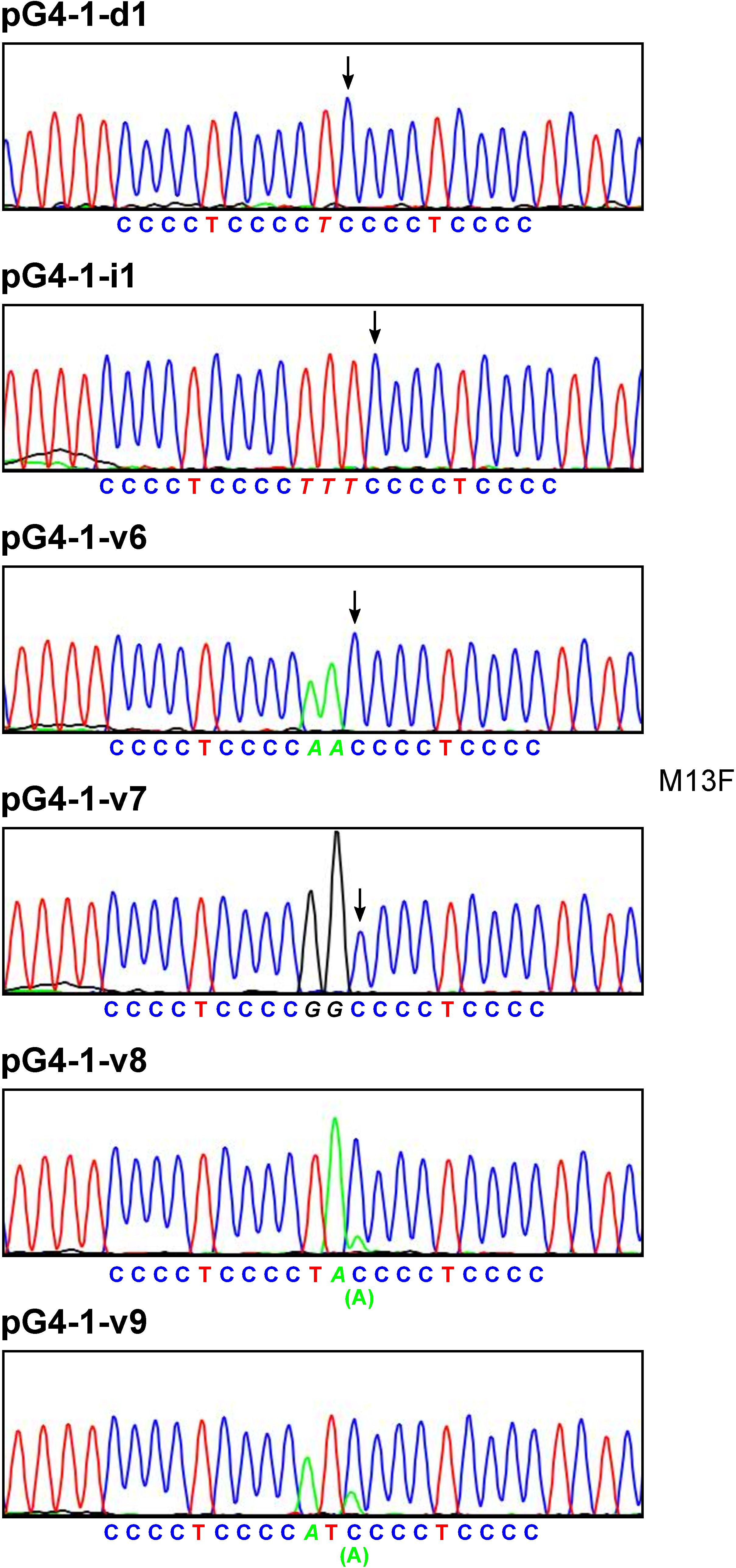

Supplement: S5 Fig — The central TT sequence was altered to change the length or composition and resulting constructs were sequenced using the M13F primer. The arrows indicate the positions corresponding to the wild-type effect. (TIF) [file pone.0279423.s006.tif]

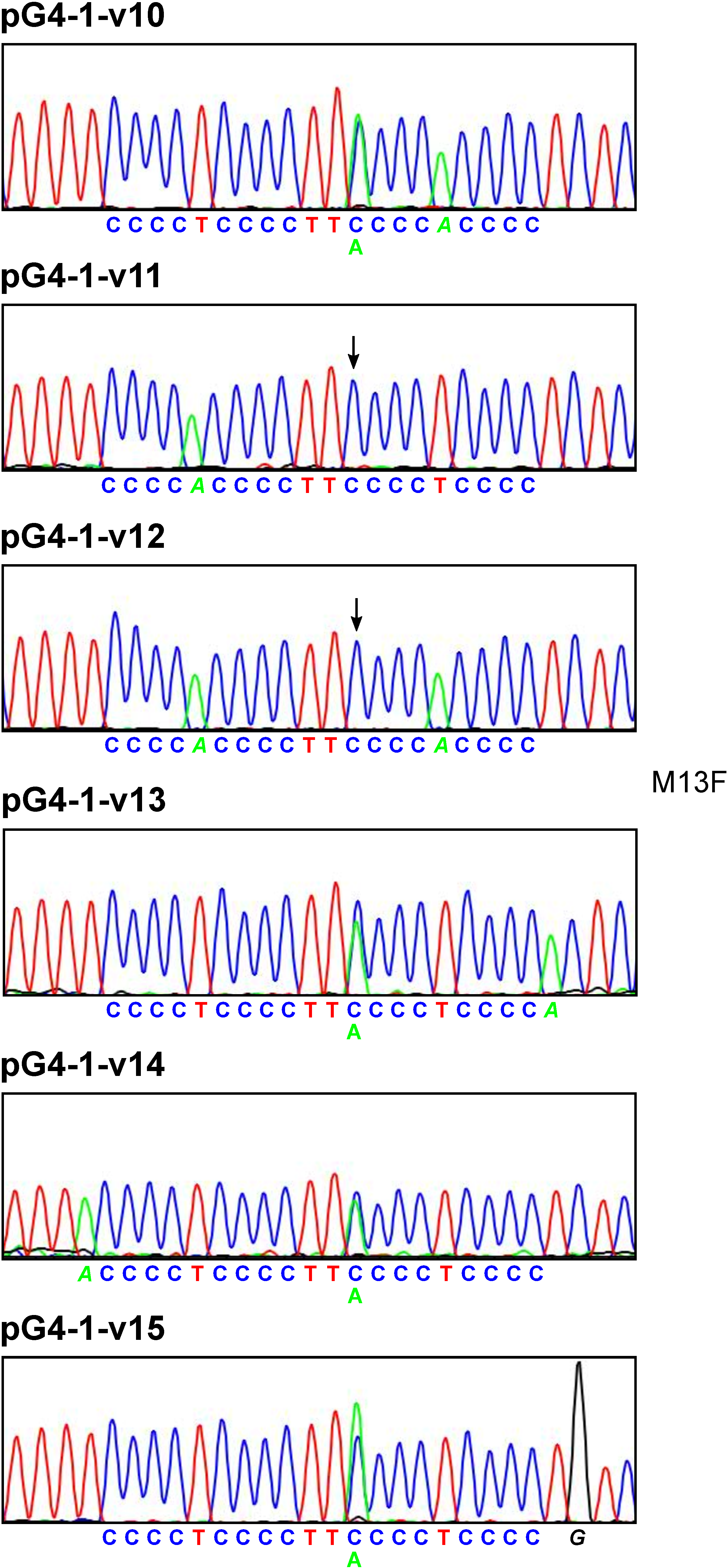

Supplement: S6 Fig — T to A changes were made in loops 1, 3, or both, and the resulting constructs were sequenced using the M13F primer. The arrows indicate the positions corresponding to the wild-type effect. (TIF) [file pone.0279423.s007.tif]

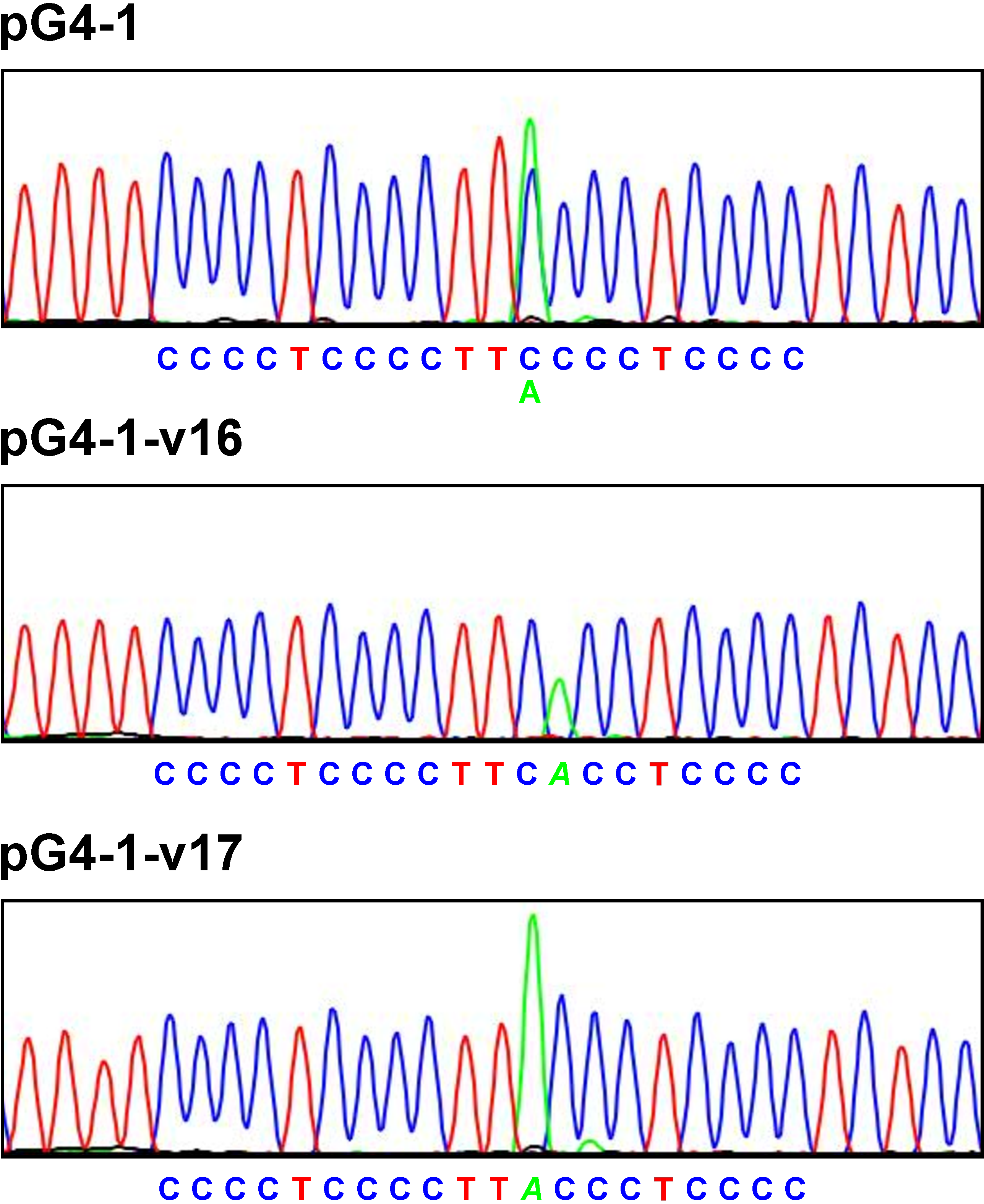

Supplement: S7 Fig — Variants were generated so that an A would be incorporated in one of two locations during sequencing (pG4-1-v16 and -v17). Tracings in comparison to pG4-1 are shown. (TIF) [file pone.0279423.s008.tif]

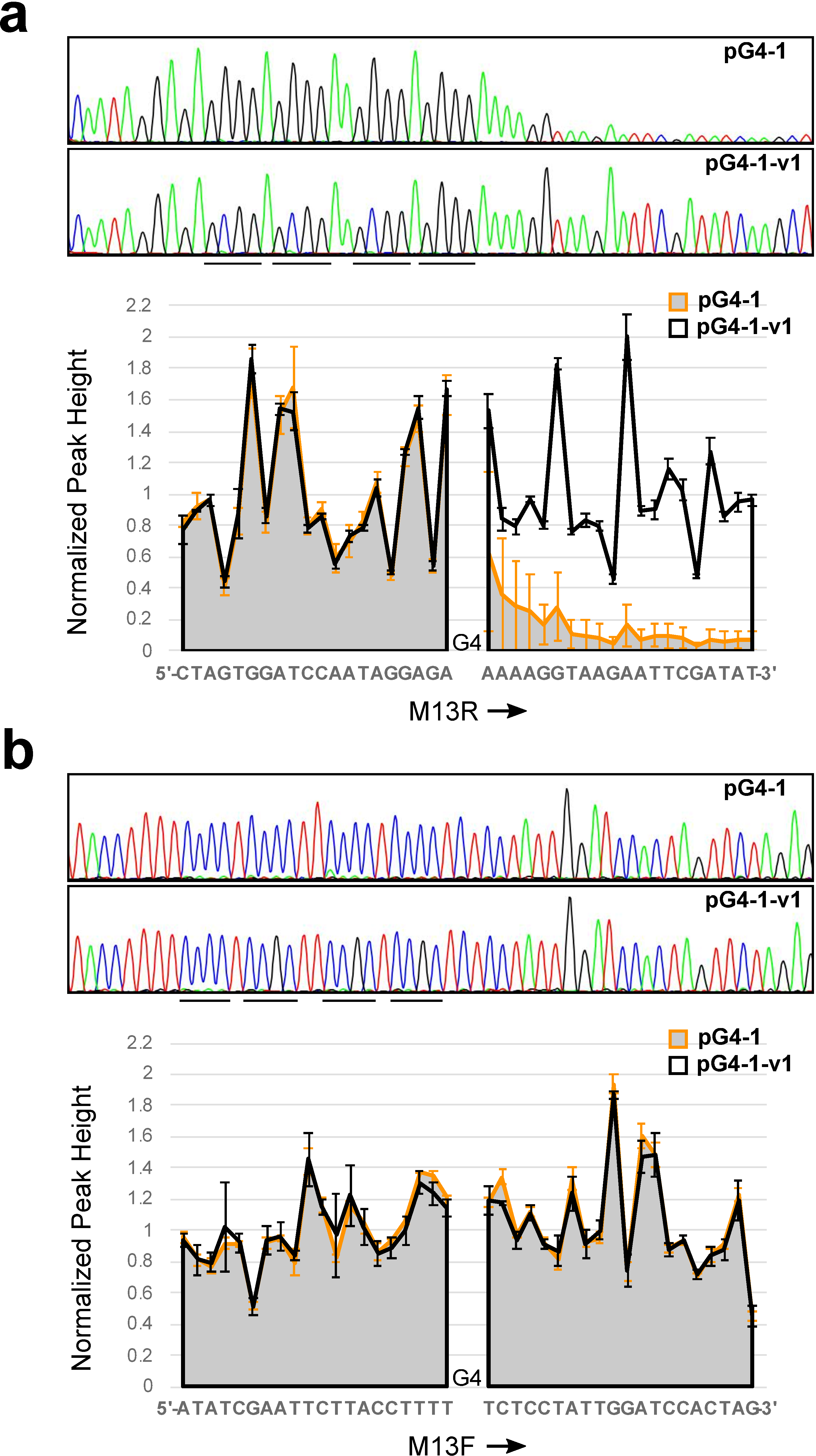

Supplement: S8 Fig — (a) Representative DNA sequence tracings (GenScript standard protocol) are shown for pG4-1 and pG4-1-v1 using the M13R primer. The lines below the tracings indicate the positions of the G4 repeats (wt or variant) The chart below indicates the average normalized peak heights before and after the G4 wt (4 runs) or variant (3 runs) sequence. Error bars indicate standard deviation. (b) As (a) but with M13F, including 4 runs for pG4-1 and 3 runs for pG4-1-v1. (TIF) [file pone.0279423.s009.tif]
