# Supplementary material for: Anomalies in dye-terminator DNA sequencing caused by a natural G-quadruplex
Source: PLoS One. 2022 Dec 27;17(12):e0279423. doi: 10.1371/journal.pone.0279423 (PMC9794070; doi:10.1371/journal.pone.0279423)
Supplement: S1 Raw images — (PDF) [file pone.0279423.s011.pdf]

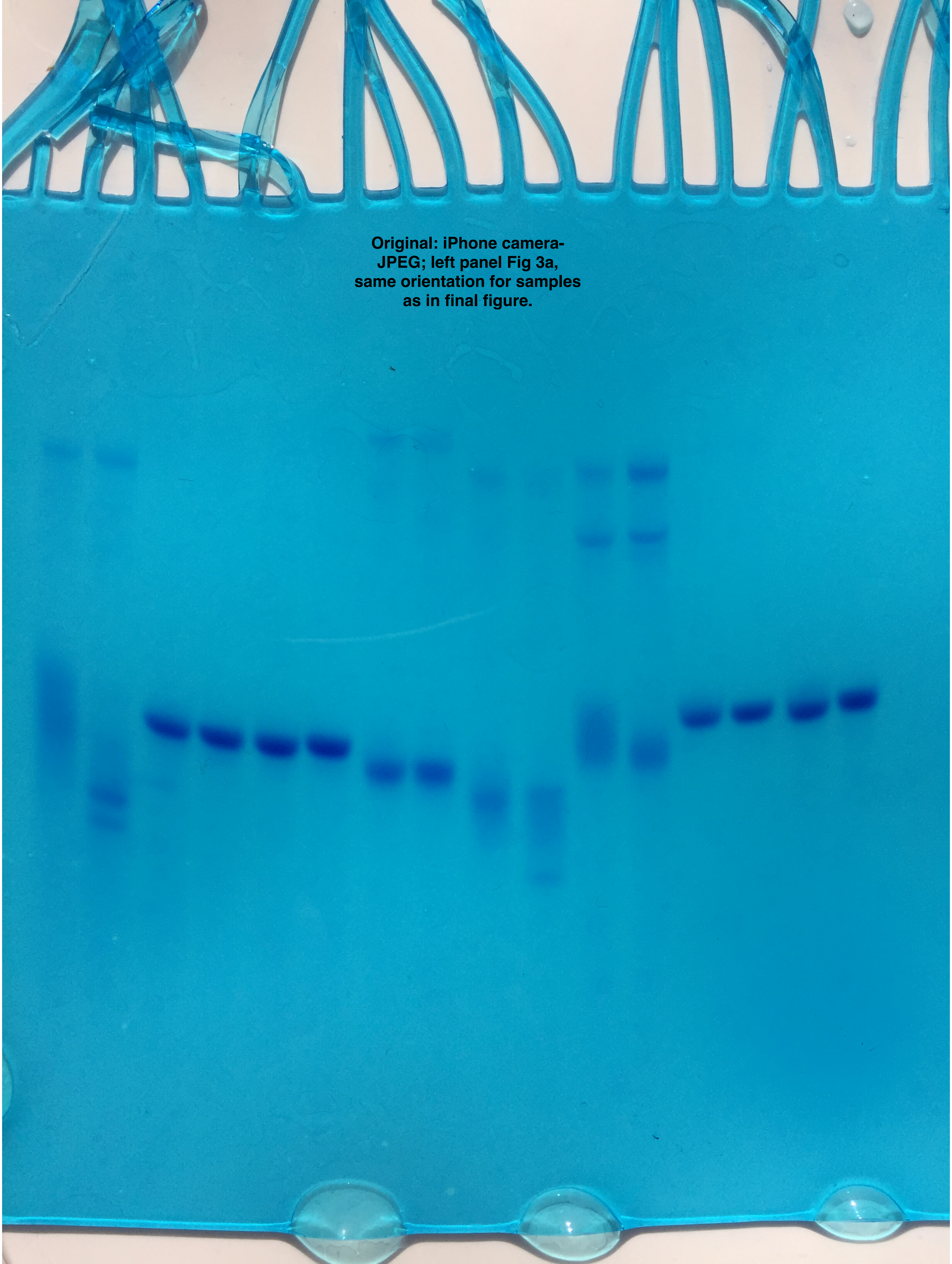
Original: iPhone camera-
JPEG; left panel Fig 3a,
same orientation for samples
as in final figure.

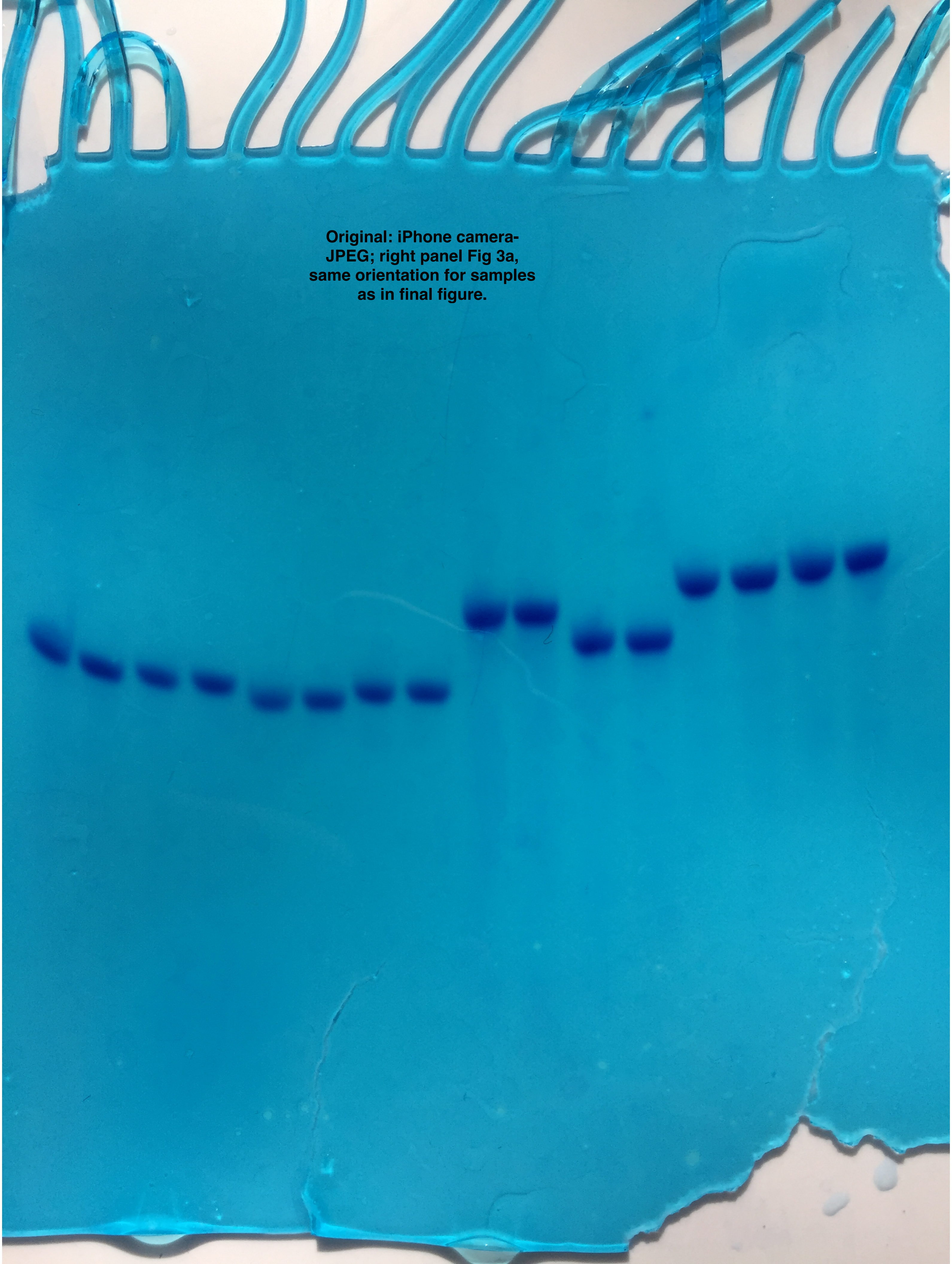
Original: iPhone camera-
JPEG; right panel Fig 3a,
same orientation for samples
as in final figure.

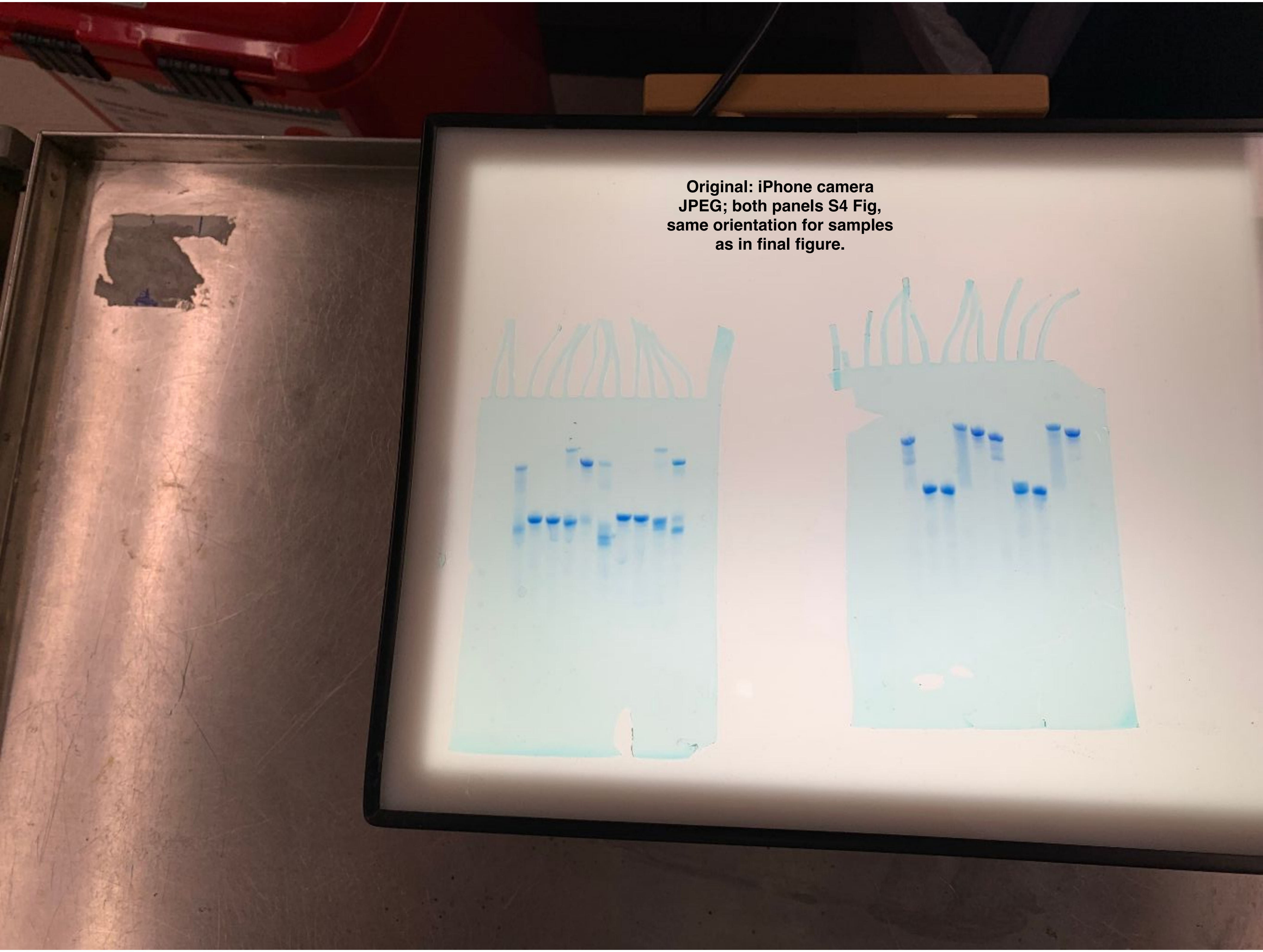
Original: iPhone camera
JPEG; both panels S4 Fig,
same orientation for samples
as in final figure.
